# Supplementary material for: Thoughts of self-harm in adolescents: Relationship with violence in the Dominican Republic
Source: PLOS Glob Public Health. 2024 Jan 8;4(1):e0002711. doi: 10.1371/journal.pgph.0002711 (PMC10773958; doi:10.1371/journal.pgph.0002711)
Supplement: S1 Checklist — (DOCX) [file pgph.0002711.s001.docx]

STROBE Statement—checklist of items that should be included in reports of observational studies

|  | Item No. | Recommendation | Page  No. | Relevant text from manuscript |
| --- | --- | --- | --- | --- |
| **Title and abstract** | 1 | (*a*) Indicate the study’s design with a commonly used term in the title or the abstract | 2 | “Cross-sectional survey data was collected at a community-based clinic from participants aged 13-20.” |
|  |  | (*b*) Provide in the abstract an informative and balanced summary of what was done and what was found | 2 | “This study aimed to examine the correlation between violence against adolescents and self-harm thoughts in the Dominican Republic (DR). Cross-sectional survey data was collected at a community-based clinic from participants aged 13-20. Participants were recruited through the clinic’s adolescent program, and verbal consent was obtained. A survey solicited information about each participant’s demographics, experiences with violence, and thoughts of harm to self or others. The survey was completed by 49 adolescents. The mean age was 16.78 (SD 2.34); 65% were female. Chi-Square, t-test, and Fisher’s exact were used to investigate the relationship between demographics, reported violence experiences and having self-harm thoughts. About half (45%) had experienced physical violence, 76% had experienced emotional violence, and 12% had experienced sexual violence. The most common perpetrators of physical and emotional violence were classmates, and the most common perpetrator of sexual violence was an ex-partner. Ten participants (20.4%) had thought about harming themselves. Self-harm thoughts were related to being female, employed, and to a higher number of experiences of physical and sexual violence. The results of this study suggest a high prevalence of both violence and self-harm thoughts in adolescents in the DR. Interventions that address physical and sexual violence against adolescents may be particularly important. Particular attention should also be paid to screening for self-harm thoughts in female-identifying adolescents. Further research is needed to better understand the relationship between violence and self-harm thoughts in adolescents in the DR.” |
| Introduction | | | |  |
| Background/rationale | 2 | Explain the scientific background and rationale for the investigation being reported | 3-5 | Violence against children is a major public health problem with an estimated one billion children having experienced sexual, physical, or emotional violence in 2016.^1^ In Latin America and the Caribbean (LAC), homicide rates are amongst the highest in the world^2^ and the leading cause of death among adolescents.^3^ This age group is susceptible not only to physical violence, but also to sexual and dating violence, emotional violence, and bullying.^4^ Up to one fifth of ever-partnered adolescent girls in LAC region have experienced interpersonal violence (IPV).^5^ Violence experienced during adolescence has been linked to a number of negative mental health outcomes such as risky behavior, substance use, depression, post-traumatic stress, and self-directed violence.^6,7,8^  There is a wealth of data supporting the idea that exposure to violence in childhood and adolescents has a long-term negative impact on mental health. One of first studies to firmly establish the negative effects of exposure to violence in childhood and adolescence on long-term health outcomes was the adverse childhood experiences (ACE) study. This study showed that ACEs, which include childhood abuse and neglect and household dysfunction, are associated with poorer mental health and overall health as adults.^9^ The majority of studies on the impacts of violence against adolescents’ on mental health have been conducted in high-income countries and outside of LAC. In the Dominican Republic (DR), it has been estimated that around 80% of adolescents have experienced at least one ACE, with the most prevalent being physical abuse and witnessing domestic violence in their household.^10^ This study also showed that in Dominican adolescents, having experienced more ACEs was associated with depression, anxiety, and dating violence perpetration and victimization. Studies across the world support the link between experiences of violence in adolescence and poor mental health. One study that took place in Guatemala showed that in children and adolescents, exposure to violence increased rates of depression and anxiety.^11^ Another study conducted in Vietnam showed that emotional violence in particular was associated with poor mental health.^12^ Studies have also shown increased rates of suicidal thoughts, depression, and post-traumatic stress in adolescents that have experienced more than one type of violence.^13,14^  Self-harm thoughts have been linked with bullying and violence against adolescents,^15^ and they are associated with increased risk of suicidal ideation and suicide completion.^16,17^ Estimates of the prevalence of self-harm thoughts and behaviors vary widely. One large meta-analysis which included estimates from many different countries estimated the lifetime prevalence of self-harm behavior to be 17%.^18^ One study from Chile found a 23% prevalence of self-harm among adolescents from low-income backgrounds.^19^ However, there is limited data on the prevalence of self-harm thoughts and behaviors in the LAC region. Self-harm thoughts and suicidal behavior have been linked to violence experienced as children or adolescents.^20^ One large study conducted in the United States found that emotional maltreatment and neglect in particular were associated with self-harm thoughts.^21^ Another study found that suicidal ideation was associated with peer victimization, sexual assault, and maltreatment by a caregiver.^22^ Peer victimization in particular has been linked to self-harm and suicidal ideation.^23,24^ One study estimated the rate of bullying in the DR at 44%,^25^ where bullying was defined as unwanted, aggressive behavior, which involves a real or perceived social power imbalance and is repeated over time.^26^  In sum, violence against adolescents is a public health problem that has major long-term impacts on both physical and mental health. There is very little information on the prevalence of violence against adolescents, self-harm thoughts, and the interaction between the two from low- and middle- income countries and from the LAC region. This study is, to our knowledge, the first to examine a relationship between violence and self-harm thoughts among adolescents in the DR. |
| Objectives | 3 | State specific objectives, including any prespecified hypotheses | 5 | This study is, to our knowledge, the first to examine a relationship between violence and self-harm thoughts among adolescents in the DR. |
| Methods | | | |  |
| Study design | 4 | Present key elements of study design early in the paper | 5-7 | Data was collected from August to October 2022. A total of 49 participants aged 13-20 and residents of La Romana, DR were recruited by educators from the clinic’s adolescent program. Verbal assent for adolescents younger than 18, and verbal consent for those 18 and older was obtained from each participant. For survey administration, questions were read by a member of the study team to small groups with no more than two to five participants at a time, as we have learned from previous studies done in this community that this is the best approach. Each adolescent filled their own paper questionnaire privately. Participants recorded their answers on the paper survey, and the research assistant checked for completeness to avoid missing data, then answers were entered into REDCap, a secure web-based survey.^27,28^ During data collection, authors involved in data collection could have identified individual participants. However, after surveys were entered into REDCap, individual participants were not able to be identified. The questionnaire administration took place in a quiet, private space and took approximately 30 to 40 minutes to complete. This study received approval from Columbia University IRB and the Dominican Republic’s National Health Bioethics Council (CONABIOS).  The questionnaire collected socio-demographic information, including age, sex, nationality, level of education, employment status, and composition of household. It also solicited information on experiences with physical, sexual, and emotional/psychological violence, on participants’ attitudes about gender and violence, and on help-seeking behaviors and thoughts of harm toward self and others.  Each type of violence - physical, sexual, and emotional/psychological - was defined for participants. They were first asked if they had ever experienced that type of violence. All participants were asked about experiences regarding specific violent acts: for example, for physical violence, participants were asked to indicate if they had experienced any of the following: been slapped, hit, or kicked; been a victim of attempted kidnapping; been stabbed; been tied up or strangled; or been threatened with a firearm. If participants reported experiencing violent acts, they were asked who perpetrated these acts of violence. Participants were asked about perpetrating violent acts against others, the types of violence committed, and who they had committed the act against.  The questionnaire also included questions about help seeking behaviors. Participants were asked if they currently felt vulnerable to violence, if they had anyone to talk to about experiences with violence, and if they knew where to go if they were a victim of violence. At the end, they were asked if they had ever thought about hurting themselves or someone else, then asked to indicate who they had thought about hurting and if they had thought about seeking help for those thoughts. Psychological support services were available at the clinic for those who were interested. |
| Setting | 5 | Describe the setting, locations, and relevant dates, including periods of recruitment, exposure, follow-up, and data collection | 5-7 | Per above |
| Participants | 6 | (*a*) *Cohort study*—Give the eligibility criteria, and the sources and methods of selection of participants. Describe methods of follow-up  *Case-control study*—Give the eligibility criteria, and the sources and methods of case ascertainment and control selection. Give the rationale for the choice of cases and controls  *Cross-sectional study*—Give the eligibility criteria, and the sources and methods of selection of participants | 5 | Data was collected from August to October 2022. A total of 49 participants aged 13-20 and residents of La Romana, DR were recruited by educators from the clinic’s adolescent program. |
|  |  | (*b*) *Cohort study*—For matched studies, give matching criteria and number of exposed and unexposed  *Case-control study*—For matched studies, give matching criteria and the number of controls per case | NA | NA |
| Variables | 7 | Clearly define all outcomes, exposures, predictors, potential confounders, and effect modifiers. Give diagnostic criteria, if applicable | 7 | We explored types of violence experienced or perpetrated by demographic characteristics. We examined whether participants experienced any type of violence perpetrated by others or committed acts of violence. We looked at self-harm thoughts, and types of violence reported by adolescents, those reporting self-harm thoughts and those who did not. |
| Data sources/ measurement | 8* | For each variable of interest, give sources of data and details of methods of assessment (measurement). Describe comparability of assessment methods if there is more than one group | 6-7 | The questionnaire collected socio-demographic information, including age, sex, nationality, level of education, employment status, and composition of household. It also solicited information on experiences with physical, sexual, and emotional/psychological violence, on participants’ attitudes about gender and violence, and on help-seeking behaviors and thoughts of harm toward self and others.  Each type of violence - physical, sexual, and emotional/psychological - was defined for participants. They were first asked if they had ever experienced that type of violence. All participants were asked about experiences regarding specific violent acts: for example, for physical violence, participants were asked to indicate if they had experienced any of the following: been slapped, hit, or kicked; been a victim of attempted kidnapping; been stabbed; been tied up or strangled; or been threatened with a firearm. If participants reported experiencing violent acts, they were asked who perpetrated these acts of violence. Participants were asked about perpetrating violent acts against others, the types of violence committed, and who they had committed the act against.  The questionnaire also included questions about help seeking behaviors. Participants were asked if they currently felt vulnerable to violence, if they had anyone to talk to about experiences with violence, and if they knew where to go if they were a victim of violence. At the end, they were asked if they had ever thought about hurting themselves or someone else, then asked to indicate who they had thought about hurting and if they had thought about seeking help for those thoughts. Psychological support services were available at the clinic for those who were interested. |
| Bias | 9 | Describe any efforts to address potential sources of bias | 5 | Each adolescent filled their own paper questionnaire privately, in order to mitigate response bias. |
| Study size | 10 | Explain how the study size was arrived at |  | This was an exploratory, pilot study to determine the frequency of violence against adolescents. As such, no sample size calculation was done since we did not formulate a hypothesis. The plan is to use the results of this pilot for a larger study. |

Continued on next page

| Quantitative variables | 11 | Explain how quantitative variables were handled in the analyses. If applicable, describe which groupings were chosen and why | 7 | Quantitative variables were handled depending on their nature: continuous variable were described depending on their distribution (mean for parametrics and median for nonparametrics), while categorical and ordinal variables were analyzed in frequencies. |
| --- | --- | --- | --- | --- |
| Statistical methods | 12 | (*a*) Describe all statistical methods, including those used to control for confounding | 7 | We began analysis with descriptive statistics of the population, to view distribution by demographic variables, and establish prevalence of suffering by each type of violence. For bivariable analysis, tests of association were used including Chi-squared test (when appropriate), Fisher’s exact test (when appropriate), and two-sample t-test (when comparing two means). A value of p < 0.05 was set for statistical significance. No multivariate analyses was done due to small sample size. |
|  |  | (*b*) Describe any methods used to examine subgroups and interactions | 7 | We began analysis with descriptive statistics of the population, to view distribution by demographic variables, and establish prevalence of suffering by each type of violence. For bivariable analysis, tests of association were used including Chi-squared test (when appropriate), Fisher’s exact test (when appropriate), and two-sample t-test (when comparing two means). A value of p < 0.05 was set for statistical significance. No multivariate analyses was done due to small sample size. |
|  |  | (*c*) Explain how missing data were addressed | 5 | No missing data was found as the research assistant checked the surveys to ensure data completion. |
|  |  | (*d*) *Cohort study*—If applicable, explain how loss to follow-up was addressed  *Case-control study*—If applicable, explain how matching of cases and controls was addressed  *Cross-sectional study*—If applicable, describe analytical methods taking account of sampling strategy | 7 | We used descriptive statistics for demographics, and tests of association (Chi square test and Fisher’s exact, when applicable) established at p<0.05 for statistical significance. |
|  |  | (*e*) Describe any sensitivity analyses |  | NA |
| Results | | | | |
| Participants | 13* | (a) Report numbers of individuals at each stage of study—eg numbers potentially eligible, examined for eligibility, confirmed eligible, included in the study, completing follow-up, and analysed | NA | Not applicable due to the peer referral sampling strategy, all adolescents that spoke with the research assistant were willing to participate, since they had been briefed on the purpose of the survey previously. |
|  |  | (b) Give reasons for non-participation at each stage |  | NA |
|  |  | (c) Consider use of a flow diagram |  | NA |
| Descriptive data | 14* | (a) Give characteristics of study participants (eg demographic, clinical, social) and information on exposures and potential confounders | 5 | 49 participants aged 13-20 years of age, living in the urban areas of La Romana, Dominican Republic. Potential confounders could include level of education, age, employment and nationality. |
|  |  | (b) Indicate number of participants with missing data for each variable of interest |  | We had not missing data in our dataset. |
|  |  | (c) *Cohort study*—Summarise follow-up time (eg, average and total amount) |  |  |
| Outcome data | 15* | *Cohort study*—Report numbers of outcome events or summary measures over time |  |  |
|  |  | *Case-control study—*Report numbers in each exposure category, or summary measures of exposure |  |  |
|  |  | *Cross-sectional study—*Report numbers of outcome events or summary measures | 9 | Of 49 participants, 18 (36.7%) reported thoughts of causing harm to themselves or others; 10 (20%) reported thoughts of self harm, of which all identified as female, 12 (24.5) ever thought of seeking help for thoughts of harm to self or others. |
| Main results | 16 | (*a*) Give unadjusted estimates and, if applicable, confounder-adjusted estimates and their precision (eg, 95% confidence interval). Make clear which confounders were adjusted for and why they were included | NA | NA |
|  |  | (*b*) Report category boundaries when continuous variables were categorized | NA | NA |
|  |  | (*c*) If relevant, consider translating estimates of relative risk into absolute risk for a meaningful time period | NA | NA |

Continued on next page

| Other analyses | 17 | Report other analyses done—eg analyses of subgroups and interactions, and sensitivity analyses | NA | NA |
| --- | --- | --- | --- | --- |
| Discussion | | | | |
| Key results | 18 | Summarise key results with reference to study objectives | 16 | Our results are consistent with previous studies which have shown higher rates of self-harm thoughts and behaviors in females.^18,29^ This study is one of the first, to our knowledge, which provides data on prevalence of thoughts of self-harm in Dominican adolescents. With a rate of nearly one third of female-identifying adolescents indicating having thought of self-harm, this indicates a great need for mental health support for this population. Agencies that provide services to this population should screen for mental health problems, especially self-harm thoughts which could indicate risk of suicide. |
| Limitations | 19 | Discuss limitations of the study, taking into account sources of potential bias or imprecision. Discuss both direction and magnitude of any potential bias | 18 and 19 | The most significant limitation in this study is its small sample size. The small sample size limited our options for what types of analysis could be conducted. In addition, since this study was done with a cross-sectional questionnaire, it was susceptible to response and recall bias. Violence tends to be under-reported because experiencing violence is traumatic and stigmatizing, especially for adolescents. The questionnaire may have also been subject to desirability bias, since they were conducted in-person and participants may have been tempted to answer questions in a way that they felt would please the interviewer. To prevent this, however, we encouraged the adolescents to fill out the surveys themselves, and each type of violence was defined by the research assistant prior to survey completion, for the sake of accuracy. |
| Interpretation | 20 | Give a cautious overall interpretation of results considering objectives, limitations, multiplicity of analyses, results from similar studies, and other relevant evidence | 16, 17 and 18 | This study was able to explore frequency of self harm thoughts and its association with violence events among adolescents. Our results reflect much of what we found in the literature, in the sense that females are more likely to report thoughts self harm, specially those who are older, unemployed and who have been victims of physical and/or sexual violence. Our results were also similar to previous reports in the DR establishing bullying and violence among classmates as a common occurrence in the adolescent population. However, measuring bullying and its effects goes beyond the scope of this study. |
| Generalisability | 21 | Discuss the generalisability (external validity) of the study results |  | The external validity of this study must be studied cautiously. We believe that our findings can be reflected in other parts of La Romana and the Dominican Republic, based on similarities with previous bullying reports in DR. However, we cannot ensure the same results in other geographical areas. |
| Other information | |  | | |
| Funding | 22 | Give the source of funding and the role of the funders for the present study and, if applicable, for the original study on which the present article is based | 20 | This project was funded by the Columbia University Vagelos College of Physicians and Surgeons Scholarly Projects program and the Friedman award.  We have no known conflicts of interest to disclose. |

*Give information separately for cases and controls in case-control studies and, if applicable, for exposed and unexposed groups in cohort and cross-sectional studies.

**Note:** An Explanation and Elaboration article discusses each checklist item and gives methodological background and published examples of transparent reporting. The STROBE checklist is best used in conjunction with this article (freely available on the Web sites of PLoS Medicine at http://www.plosmedicine.org/, Annals of Internal Medicine at http://www.annals.org/, and Epidemiology at http://www.epidem.com/). Information on the STROBE Initiative is available at www.strobe-statement.org.
