# Supplementary material for: Thoughts of self-harm in adolescents: Relationship with violence in the Dominican Republic
Source: PLOS Glob Public Health. 2024 Jan 8;4(1):e0002711. doi: 10.1371/journal.pgph.0002711 (PMC10773958; doi:10.1371/journal.pgph.0002711)
Supplement: S1 Dataset — (PDF) [file pgph.0002711.s003.pdf]

AlcanceYTiposDeViole_DATA_2022-11-16_1448 (1) (2)

record_id	redcap_survey_identifier	form_1_timestamp	rural	edad	sexo	sexo_otro	nacion	nac_otro	curso	asistencia	abandono	anos_ab	razon_ab___1	razon_ab___2	razon_ab___3	razon_ab___4	razon_ab___5	razon_ab___88	razon_ab___89	otro_razon_ab	vive___1	vive___2	vive___3	vive___4	vive___5	vive___6	vive___88	vive___89	otro_vive
1		2022-08-17 22:30:28	1	20	2		1		5	1	2		0	0	0	0	0	0	0		0	1	0	0	0	0	0	0	
2		2022-08-17 22:39:15	1	18	2		1		5	1	2		0	0	0	0	0	0	0		0	0	0	0	0	1	0	0	Sola
3		2022-08-23 20:43:35	1	18	1		1		4	1	2		0	0	0	0	0	0	0		0	0	1	0	0	0	0	0	
4		2022-08-23 20:49:12	1	15	2		1		3	1	2		0	0	0	0	0	0	0		1	0	0	0	0	0	0	0	
5		2022-08-23 20:58:07	1	17	1		1		4	2	2		0	0	0	0	0	0	0		0	1	0	0	0	0	0	0	
6		2022-08-23 21:05:03	0	18	2		1		4	1	2		0	0	0	0	0	0	0		0	1	0	0	0	0	0	0	
7		2022-08-24 20:42:35	1	20	2		1		3	88	2		0	0	0	0	0	0	0		1	0	0	0	0	0	0	0	
8		2022-08-24 20:51:11	1	19	1		1		4	2	2		0	0	0	0	0	0	0		0	0	0	1	0	0	0	0	
9		2022-08-24 20:56:25	1	17	1		1		4	2	88		0	0	0	0	0	0	0		0	1	0	0	0	0	0	0	
10		2022-09-05 16:09:11	1	15	2		1		3	2	2		0	0	0	0	0	0	0		0	1	0	0	0	0	0	0	
11		2022-09-05 16:12:38	1	17	2		1		4	2	2		0	0	0	0	0	0	0		0	1	0	0	0	0	0	0	
12		2022-09-05 16:16:16	0	16	2		1		3	2	2		0	0	0	0	0	0	0		0	0	1	0	0	0	0	0	
13		2022-09-05 16:19:39	1	19	1		1		4	1	2		0	0	0	0	0	0	0		1	0	0	0	0	0	0	0	
14		2022-09-05 16:24:35	1	16	2		1		3	2	2		0	0	0	0	0	0	0		1	0	0	0	0	0	0	0	
15		2022-09-06 17:06:26	1	17	1		1		4	2	2		0	0	0	0	0	0	0		0	0	1	0	0	0	0	0	
16		2022-09-06 17:10:46	1	15	1		1		2	1	1	10	0	1	0	0	0	0	0		0	1	1	0	0	0	0	0	
17		2022-09-06 17:14:28	1	16	2		1		3	1	2		0	0	0	0	0	0	0		1	0	0	0	0	0	0	0	
18		2022-09-06 17:18:54	1	15	2		1		3	1	2		0	0	0	0	0	0	0		0	0	1	0	0	0	0	0	
19		2022-09-06 17:22:35	1	15	2		1		3	1	2		0	0	0	0	0	0	0		0	1	1	0	0	0	0	0	
20		2022-09-21 20:33:08	1	16	2		1		3	1	2		0	0	0	0	0	0	0		0	0	0	1	0	0	0	0	
21		2022-09-21 20:39:20	1	17	2		1		4	1	2		0	0	0	0	0	0	0		0	0	0	0	0	1	0	0	pareja
22		2022-09-23 23:12:09	1	20	2		1		4	2	2		0	0	0	0	0	0	0		1	0	0	0	0	0	0	0	
23		2022-09-23 23:42:19	1	20	2		1		2	2	1	12	0	0	0	0	1	0	0	tenia hijo	0	0	0	0	0	1	0	0	hijo
24		2022-09-23 23:47:09	1	17	2		1		4	2	2		0	0	0	0	0	0	0		0	0	1	0	0	0	0	0	
25		2022-09-23 23:50:34	1	19	2		1		5	2	2		0	0	0	0	0	0	0		0	1	0	0	0	0	0	0	
26		2022-09-26 16:51:40	1	13	1		1		3	1	2		0	0	0	0	0	0	0		0	1	0	0	0	0	0	0	
27		2022-09-26 16:56:46	1	13	2		1		3	1	2		0	0	0	0	0	0	0		0	0	0	1	0	0	0	0	
28		2022-09-26 17:01:05	1	16	1		1		3	1	2		0	0	0	0	0	0	0		1	0	0	0	0	0	0	0	
29		2022-09-26 17:11:17	1	15	2		1		3	1	2		0	0	0	0	0	0	0		0	1	1	0	0	0	0	0	
30		2022-09-26 17:28:37	1	13	2		1		1	2	1	11	0	0	0	0	1	0	0	no me inscribieron en la escuela	0	1	1	0	1	0	0	0	
31		2022-09-26 17:31:37	1	13	2		1		3	1	2		0	0	0	0	0	0	0		0	1	1	1	0	0	0	0	
32		2022-09-26 17:34:36	1	14	1		1		1	2	2		0	0	0	0	0	0	0		0	1	0	0	0	0	0	0	
33		2022-09-26 17:40:47	1	16	2		1		3	1	2		0	0	0	0	0	0	0		0	1	0	0	0	0	0	0	
34		2022-09-26 17:47:23	1	13	1		1		3	1	2		0	0	0	0	0	0	0		0	1	1	0	0	0	0	0	
35		2022-09-26 17:51:08	1	20	2		1		4	2	2		0	0	0	0	0	0	0		0	1	0	0	0	0	0	0	
36		2022-09-26 17:56:05	1	20	1		1		3	1	2		0	0	0	0	0	0	0		0	0	0	0	0	1	0	0	solo
37		2022-09-26 17:59:07	1	14	1		1		3	1	2		0	0	0	0	0	0	0		1	0	0	0	0	0	0	0	
38		2022-09-26 18:06:02	1	13	2		1		2	1	2		0	0	0	0	0	0	0		0	0	0	1	0	0	0	0	
39		2022-09-26 20:11:17	1	19	1		1		4	2	1	17	1	0	0	0	0	0	0		0	0	0	1	0	0	0	0	
40		2022-09-26 20:18:09	1	15	89		1		4	2	2		0	0	0	0	0	0	0		1	0	1	0	0	0	0	0	
41		2022-09-26 22:01:18	1	20	2		1		4	2	2		0	0	0	0	0	0	0		0	1	0	0	0	0	0	0	
42		2022-09-26 22:06:14	1	14	2		1		2	2	1	13	0	0	0	0	0	0	1		1	0	0	0	0	0	0	0	
43		2022-09-26 22:09:36	1	16	2		1		4	2	2		0	0	0	0	0	0	0		1	0	0	0	0	0	0	0	
44		2022-09-26 22:14:03	1	17	2		1		1	1	1	2	0	0	0	0	1	0	0		1	0	0	0	0	0	0	0	
45		2022-09-26 22:18:05	1	20	2		1		4	2	2		0	0	0	0	0	0	0		0	1	0	0	0	0	0	0	
46		2022-09-26 22:21:24	1	20	2		1		4	1	1	15	0	0	0	1	0	0	0		0	0	1	0	0	0	0	0	
47		2022-10-03 17:17:46	1	19	1		1		4	1	1	17	0	0	0	0	1	0	0	embarazada	0	0	0	0	0	1	0	0	esposo
48		2022-10-04 18:09:37	1	18	1		1		3	2	2		0	0	0	0	0	0	0		1	0	0	0	0	0	0	0	
49		2022-10-04 18:14:53	1	19	2		1		3	2	1	18	1	0	0	0	0	0	0		0	1	0	0	0	0	0	0	

trabajo	tipo_trabajo	otro_tipo	fisica_1	fisica_2	fisica_3___1	fisica_3___2	fisica_3___3	fisica_3___4	fisica_3___5	fisica_3___6	fisica_3___88	fisica_4___1	fisica_4___2	fisica_4___3	fisica_4___4	fisica_4___5	fisica_4___6	fisica_4___7	fisica_4___88	fisica_4___89	fisica_4___90	otro_fisica_4	fisica_5___1	fisica_5___2	fisica_5___3	fisica_5___4	fisica_5___5	fisica_5___6
1	3		2	1	1	0	0	0	1	0	0	0	0	0	0	0	0	0	0	1	0		0	0	0	0	0	1
1	1		2	1	0	0	0	0	1	0	0	0	0	0	0	0	0	0	0	1	0		0	0	0	0	0	1
2			2	1	0	0	0	0	1	0	0	0	0	0	0	0	0	0	1	0	0		1	0	0	0	0	0
2			2	1	1	0	0	0	0	0	0	0	0	1	0	0	0	0	0	0	0		1	0	0	0	0	0
2			1	1	1	0	0	0	0	0	0	0	0	0	0	0	1	0	0	0	0		0	0	0	0	0	0
2			2	88	1	0	0	0	0	0	0	0	0	0	1	0	0	0	0	0	0		1	0	0	0	0	0
2			1	1	0	0	0	0	0	0	1	0	0	0	0	0	0	0	0	0	0		0	0	0	0	0	0
2			1	1	0	0	0	0	0	0	1	0	0	0	0	0	0	0	0	0	0		0	0	0	0	0	1
2			88	1	0	0	0	0	0	0	1	0	0	0	0	0	0	0	0	0	0		0	0	0	0	0	0
2			1		0	0	0	0	0	0	1	0	0	0	0	0	0	0	0	0	0		0	0	0	0	0	1
2			1		0	0	0	0	0	0	1	0	0	0	0	0	0	0	0	0	0		0	0	0	0	0	1
2			1		0	0	0	0	0	1	0	0	0	0	0	0	0	0	0	0	0		0	0	0	0	0	1
1	1		1		0	0	0	0	0	1	0	0	0	0	0	0	0	0	0	0	0		0	0	0	0	0	1
2			1		0	0	0	0	0	0	1	0	0	0	0	0	0	0	0	0	0		0	0	0	0	0	0
2			2	88	1	0	0	0	0	0	0	0	0	0	0	0	0	0	0	1	0		1	0	0	0	0	0
2			2	2	1	0	0	0	1	0	0	0	0	0	0	0	1	0	0	0	1		1	0	0	0	1	0
2			1		0	0	0	0	0	1	0	0	0	0	0	0	0	0	0	0	0		0	0	0	0	0	0
1	1		2	1	1	0	0	0	0	0	0	0	0	0	1	0	0	0	0	0	0		1	0	0	1	0	0
2			1		0	0	0	0	0	1	0	0	0	0	0	0	0	0	0	0	0		0	0	0	0	0	1
2			1		0	0	0	0	0	1	0	0	0	0	0	0	0	0	0	0	0		0	0	0	0	0	0
2			1		1	0	0	0	0	0	0	0	0	0	0	0	0	0	1	0	1		1	0	0	0	0	0
1	1		1		0	0	0	0	0	1	0	0	0	0	0	0	0	0	0	0	0		0	0	0	0	0	1
1	1		2	1	0	0	0	0	1	0	0	0	0	0	0	0	0	0	0	1	0		1	0	0	0	0	0
2			1		0	0	0	0	0	1	0	0	0	0	0	0	0	0	0	0	0		0	0	0	0	0	1
1	1		89		0	0	0	0	0	1	0	0	0	0	0	0	0	0	0	0	0		0	0	0	0	0	1
2			1		1	0	0	0	0	1	0	0	0	1	0	0	0	0	0	0	0		0	0	0	0	0	1
2			2	1	0	0	0	0	0	1	0	0	0	0	0	0	0	0	0	0	0		0	0	0	0	0	1
2			1		0	0	0	0	0	1	0	0	0	0	0	0	0	0	0	0	0		0	0	0	0	0	1
2			1		0	0	0	0	0	1	0	0	0	0	0	0	0	0	0	0	0		0	0	0	0	0	1
2			1		0	0	0	0	0	1	0	0	0	0	0	0	0	0	0	0	0		1	0	0	0	0	0
2			2	2	1	0	0	0	0	0	0	0	0	0	0	0	1	0	0	0	0		1	0	0	0	0	0
2			1		0	0	0	0	0	1	0	0	0	0	0	0	0	0	0	0	0		0	0	0	0	0	1
2			1		1	0	0	0	0	0	0	0	0	0	1	0	0	0	0	0	0		0	0	0	0	0	1
2			1		0	0	0	0	0	1	0	0	0	0	0	0	0	0	0	0	0		0	0	0	0	0	1
2			2	1	1	0	0	0	0	0	0	0	0	1	0	0	0	0	0	0	0		0	0	0	0	0	1
1	2		1		0	0	0	0	1	0	0	0	0	0	0	0	0	0	0	0	1		1	0	0	0	0	0
2			1		0	0	0	0	0	1	0	0	0	0	0	0	0	0	0	0	0		1	0	0	0	0	0
2			1		0	0	0	0	0	0	1	0	0	0	0	0	0	0	0	0	0		0	0	0	0	0	1
1	1		1		1	0	0	0	0	0	0	0	0	0	0	0	1	0	0	0	0		1	0	0	0	0	0
1	2		1		1	0	0	0	0	0	0	0	0	0	0	0	1	0	0	1	0		1	0	0	0	0	0
2			1		0	0	0	0	0	1	0	0	0	0	0	0	0	0	0	0	0		0	0	0	0	0	1
2			1		0	0	0	0	0	1	0	0	0	0	0	0	0	0	0	0	0		0	0	0	0	0	1
2			1		0	0	0	0	0	1	0	0	0	0	0	0	0	0	0	0	0		0	0	0	0	0	1
2			1		0	0	0	0	0	1	0	0	0	0	0	0	0	0	0	0	0		0	0	0	0	0	1
1	1		2	1	1	0	0	0	0	0	0	0	1	0	0	0	0	0	0	0	0		1	0	0	0	0	0
2			1		1	0	0	0	0	0	0	0	0	0	0	0	1	0	0	0	0		1	0	0	0	0	0
1	2		1		0	0	0	0	0	1	0	0	0	0	0	0	0	0	0	0	0		0	0	0	0	0	1
2			2	1	0	0	0	0	1	0	0	0	1	0	0	0	0	0	0	0	0		0	0	1	0	0	0
2			2	2	1	0	0	0	0	0	0	0	1	0	0	0	0	0	0	0	0		0	0	0	0	0	1

fisica_5___88	fisica_6___1	fisica_6___2	fisica_6___3	fisica_6___4	fisica_6___5	fisica_6___6	fisica_6___7	fisica_6___88	fisica_6___89	fisica_6___90	otro_fisica_6	emocional_1	emocional_2	emocional_3___1	emocional_3___2	emocional_3___3	emocional_3___4	emocional_3___5	emocional_3___88	emocional_4___1	emocional_4___2	emocional_4___3	emocional_4___4
0	0	0	0	0	0	0	0	0	0	0		2	2	1	1	1	0	0	0	1	1	0	1
0	0	0	0	0	0	0	0	0	0	0		2	1	0	1	0	0	0	0	0	0	0	1
0	0	0	0	0	0	1	0	0	0	0		2	89	1	1	0	0	0	0	0	0	0	1
0	0	0	0	0	0	1	0	0	0	0		2	2	1	0	0	0	0	0	0	0	0	1
1	0	0	0	0	0	0	0	0	0	0		1	1	0	0	0	0	0	1	0	0	0	0
0	0	0	0	1	0	0	0	0	0	0		1	1	0	1	0	0	0	0	0	0	0	1
1	0	0	0	0	0	0	0	0	0	0		1	89	0	0	1	0	0	0	1	0	0	0
0	0	0	0	0	0	0	0	0	0	0		1	1	1	1	0	0	0	0	0	1	1	0
1	0	0	0	0	0	0	0	0	0	0		88	1	0	0	0	0	0	1	0	0	0	0
0	0	0	0	0	0	0	0	0	0	0		1		1	0	0	0	0	0	0	0	0	0
0	0	0	0	0	0	0	0	0	0	0		1		1	0	0	0	0	0	0	0	0	0
0	0	0	0	0	0	0	0	0	0	0		1		0	0	0	0	0	1	0	0	0	0
0	0	0	0	0	0	0	0	0	0	0		1		0	0	0	0	1	0	0	0	0	0
1	0	0	0	0	0	0	0	0	0	0		1		0	0	0	0	0	1	0	0	0	0
0	0	0	0	0	0	0	0	0	1	0		1		0	0	0	0	1	0	0	0	0	0
0	0	0	0	0	0	1	0	0	0	1		2	2	1	0	0	0	0	0	0	0	0	0
1	0	0	0	0	0	0	0	0	0	0		1		1	0	0	0	0	0	0	0	0	0
0	0	0	0	0	0	0	0	1	0	0		2	1	1	1	0	1	0	0	0	0	0	1
0	0	0	0	0	0	0	0	0	0	0		1		1	0	0	0	0	0	0	1	1	0
1	0	0	0	0	0	0	0	0	0	0		2	1	1	1	0	1	0	0	0	1	0	0
0	0	0	0	0	0	0	0	1	0	1		1		1	0	1	1	0	0	0	1	0	0
0	0	0	0	0	0	0	0	0	0	0		1		0	0	0	0	1	0	0	0	0	0
0	0	1	0	0	0	0	0	0	0	0		2	1	1	1	0	0	0	0	0	1	0	0
0	0	0	0	0	0	0	0	0	0	0		1		1	0	0	0	0	0	0	0	0	0
0	0	0	0	0	0	0	0	0	0	0		2	1	1	0	0	0	0	0	0	0	1	0
0	0	0	0	0	0	0	0	0	0	0		2	2	1	0	0	0	0	0	0	0	1	0
0	0	0	0	0	0	0	0	0	0	0		2	1	1	0	0	0	0	0	0	0	0	0
0	0	0	0	0	0	0	0	0	0	0		2	1	1	0	0	0	0	0	0	0	0	0
0	0	0	0	0	0	0	0	0	0	0		2	1	0	0	0	0	1	0	0	0	0	0
0	0	0	0	0	0	0	0	1	0	0		1		1	0	1	0	0	0	0	0	0	0
0	0	0	0	0	0	1	0	0	0	0		1		1	0	0	0	0	0	0	0	0	0
0	0	0	0	0	0	0	0	0	0	0		1		0	0	0	0	1	0	0	0	0	0
0	0	0	0	0	0	0	0	0	0	0		89		1	1	0	1	0	0	0	0	0	0
0	0	0	0	0	0	0	0	0	0	0		1		1	0	1	0	0	0	0	0	0	0
0	0	0	0	0	0	0	0	0	0	0		2	2	1	0	0	0	0	0	1	0	0	0
0	0	0	0	0	0	1	0	0	0	0		2	1	1	1	0	0	0	0	0	0	0	0
0	0	0	0	1	0	1	0	0	0	0		1		1	0	1	0	0	0	0	0	0	0
0	0	0	0	0	0	0	0	0	0	0		1		0	0	0	0	1	0	0	0	0	0
0	0	0	0	0	0	1	0	0	0	0		2	88	1	0	0	0	0	0	0	0	0	0
0	0	0	0	0	0	0	0	1	0	0		1		1	1	0	0	0	0	0	0	0	0
0	0	0	0	0	0	0	0	0	0	0		1		0	0	0	0	1	0	0	0	0	0
0	0	0	0	0	0	0	0	0	0	0		1		0	0	0	0	1	0	0	0	0	0
0	0	0	0	0	0	0	0	0	0	0		1		1	0	0	0	0	0	0	0	0	1
0	0	0	0	0	0	0	0	0	0	0		2	1	0	0	0	1	0	0	0	0	0	0
0	0	1	0	0	0	0	0	0	0	0		2	1	1	0	0	0	0	0	0	1	0	0
0	0	0	0	0	0	1	0	0	0	0		2	1	1	1	1	1	0	0	0	0	0	0
0	0	0	0	0	0	0	0	0	0	0		2	2	1	0	0	0	0	0	0	1	0	0
0	0	0	0	0	0	1	0	0	0	0		2	2	1	0	0	0	0	0	0	1	0	0
0	0	0	0	0	0	0	0	0	0	0		2	2	0	1	1	0	0	0	0	1	0	0

emocional_4___5	emocional_4___6	emocional_4___7	emocional_4___88	emocional_4___89	emocional_4___90	otro_emoc_4	emocional_5___1	emocional_5___2	emocional_5___3	emocional_5___4	emocional_5___5	emocional_5___88	emocional_6___1	emocional_6___2	emocional_6___3	emocional_6___4	emocional_6___5	emocional_6___6	emocional_6___7
0	1	0	0	1	0		1	0	0	0	0	0	1	1	0	1	0	0	0
0	0	0	0	0	0		0	0	0	0	1	0	0	0	0	0	0	0	0
0	0	0	0	0	0		1	1	1	0	0	0	0	0	0	0	0	1	0
0	1	0	0	0	0		1	0	0	0	0	0	0	0	0	0	0	1	0
0	0	0	0	0	0		1	0	0	0	0	0	0	0	0	0	0	0	0
0	0	0	0	0	0		1	1	0	1	0	0	0	0	0	1	1	0	0
0	0	0	0	0	0		1	0	0	0	0	0	0	0	0	0	0	1	0
0	0	0	0	0	0		1	1	0	0	0	0	0	1	0	0	0	0	0
0	0	0	0	0	0		0	0	0	0	0	1	0	0	0	0	0	0	0
0	0	0	1	0	0		1	0	0	0	0	0	0	0	0	0	0	0	0
0	0	0	1	0	0		1	0	0	0	0	0	0	0	0	0	0	0	0
0	0	0	0	0	0		0	0	0	0	0	1	0	0	0	0	0	0	0
0	0	0	0	0	0		0	0	0	0	1	0	0	0	0	0	0	0	0
0	0	0	0	0	0		1	0	0	0	0	0	0	0	0	0	0	0	0
0	0	0	0	0	0		0	0	0	0	1	0	0	0	0	0	0	0	0
0	1	0	0	0	1		0	1	0	0	0	0	0	0	0	0	0	1	0
0	1	0	0	0	0		1	0	0	0	0	0	0	0	0	0	0	1	0
0	0	0	0	0	0		1	1	1	0	0	0	0	0	0	0	0	1	0
0	0	0	0	0	0		0	0	1	0	0	0	0	0	1	0	0	0	1
0	1	0	0	0	0		1	0	0	0	0	0	0	1	0	0	0	0	0
0	0	0	0	0	0		1	1	0	0	0	0	0	0	0	0	0	1	0
0	0	0	0	0	0		0	0	0	0	1	0	0	0	0	0	0	0	0
0	0	0	0	0	0		0	0	0	0	0	1	0	0	0	0	0	0	0
0	0	0	1	0	0		0	0	0	0	1	0	0	0	0	0	0	0	0
0	0	0	0	0	0		0	0	0	0	1	0	0	0	0	0	0	0	0
0	0	0	0	0	0		0	0	0	0	1	0	0	0	0	0	0	0	0
0	1	0	0	0	0		0	0	0	0	1	0	0	0	0	0	0	0	0
0	0	1	0	0	0		1	0	0	0	0	0	0	0	0	0	0	1	0
0	0	0	0	0	0		0	0	0	0	1	0	0	0	0	0	0	0	0
0	1	0	0	0	0		1	0	0	0	0	0	0	0	0	0	0	1	0
0	1	0	0	0	0		0	0	0	0	1	0	0	0	0	0	0	0	0
0	0	0	0	0	0		0	0	0	0	1	0	0	0	0	0	0	0	0
0	0	0	1	0	0		1	0	0	0	0	0	0	0	0	0	0	0	0
0	0	0	1	0	0		0	0	0	0	1	0	0	0	0	0	0	0	0
0	0	0	0	0	0		0	0	0	0	1	0	0	0	0	0	0	0	0
0	0	0	1	0	0		1	0	1	0	0	0	0	0	0	0	0	0	0
0	1	0	0	0	0		0	0	0	0	0	1	0	0	0	0	0	0	0
0	0	0	0	0	0		0	0	0	0	1	0	0	0	0	0	0	0	0
0	1	0	0	0	0		1	0	0	0	0	0	0	0	0	0	0	1	0
0	0	0	1	0	0		1	0	0	0	0	0	0	0	0	0	0	0	0
0	0	0	0	0	0		0	0	0	0	1	0	0	0	0	0	0	0	0
0	0	0	0	0	0		0	0	0	0	0	1	0	0	0	0	0	0	0
0	1	0	0	0	0		0	0	0	0	1	0	0	0	0	0	0	0	0
0	0	0	0	1	0		1	0	0	0	0	0	0	0	0	1	0	0	0
0	0	0	0	0	0		1	0	0	0	0	0	0	0	0	1	0	0	0
0	1	0	0	0	0		1	0	0	0	0	0	0	0	0	0	0	1	0
0	0	0	0	0	0		0	0	0	0	1	0	0	0	0	0	0	0	0
0	0	0	0	0	0		0	0	0	0	1	0	0	0	0	0	0	0	0
0	0	0	0	0	1	amigo	1	0	0	0	0	0	0	0	0	0	0	0	0

emocional_6___88	emocional_6___89	emocional_6___90	otro_emoc_6	sexual_1	sexual_2	sexual_3___1	sexual_3___2	sexual_3___3	sexual_3___4	sexual_3___5	sexual_3___6	sexual_3___88	sexual_4___1	sexual_4___2	sexual_4___3	sexual_4___4	sexual_4___5	sexual_4___6	sexual_4___7	sexual_4___88	sexual_4___89	sexual_4___90	otro_sex_4	sexual_5___1
0	0	0		2	1	1	0	1	0	1	0	0	0	0	0	0	0	0	0	0	0	1	"Amigo"	0
0	0	0		1		0	0	0	0	0	0	0	0	0	0	0	0	0	0	0	0	0		0
0	0	0		1		0	0	0	0	0	0	1	0	0	0	0	0	0	0	0	0	0		0
0	0	0		1		0	0	0	0	0	0	1	0	0	0	0	0	0	0	0	0	0		0
0	1	0		1		0	0	0	0	0	0	1	0	0	0	0	0	0	0	0	0	0		0
0	0	0		1		0	0	0	0	0	0	1	0	0	0	0	0	0	0	0	0	0		0
0	0	0		1		0	1	0	0	0	0	0	1	0	0	0	0	0	0	0	0	0		0
0	0	0		1		0	0	0	0	0	0	1	0	0	0	0	0	0	0	0	0	0		0
0	0	0		88		0	0	0	0	0	0	1	0	0	0	0	0	0	0	0	0	0		0
1	0	0		1		0	0	0	0	0	1	0	0	0	0	0	0	0	0	0	0	0		0
1	0	0		1		0	0	0	0	0	1	0	0	0	0	0	0	0	0	0	0	0		0
0	0	0		1		0	0	0	0	0	1	0	0	0	0	0	0	0	0	0	0	0		0
0	0	0		1		0	0	0	0	0	1	0	0	0	0	0	0	0	0	0	0	0		0
1	0	0		1		0	0	0	0	0	1	0	0	0	0	0	0	0	0	0	0	0		0
0	0	0		1		0	0	0	0	0	1	0	0	0	0	0	0	0	0	0	0	0		0
0	0	0		1		0	0	0	0	0	1	0	0	0	0	0	0	0	0	0	0	0		0
0	0	0		1		0	0	0	0	0	1	0	0	0	0	0	0	0	0	0	0	0		0
0	0	0		2	1	0	0	0	0	1	0	0	0	0	0	0	0	0	0	1	0	0		0
0	0	0		1		0	0	0	0	0	1	0	0	0	0	0	0	0	0	0	0	0		0
0	0	0		1		0	0	0	0	0	0	1	0	0	0	0	0	0	0	0	0	0		0
0	0	1		1		0	0	0	0	0	1	0	0	0	0	0	0	0	0	0	0	0		0
0	0	0		1		0	0	0	0	0	1	0	0	0	0	0	0	0	0	0	0	0		0
0	0	0		2	1	1	0	0	0	0	0	0	0	0	0	0	0	0	0	1	0	0		1
0	0	0		1		0	0	0	0	0	1	0	0	0	0	0	0	0	0	0	0	0		0
0	0	0		1		0	0	0	0	0	1	0	0	0	0	0	0	0	0	0	0	0		0
0	0	0		1		0	0	0	0	0	1	0	0	0	0	0	0	0	0	0	0	0		0
0	0	0		1		0	0	0	0	0	1	0	0	0	0	0	0	0	0	0	0	0		0
0	0	0		1		0	0	0	0	0	1	0	0	0	0	0	0	0	0	0	0	0		0
0	0	0		1		0	0	0	0	0	1	0	0	0	0	0	0	0	0	0	0	0		0
0	0	0		88		0	0	0	0	0	0	1	0	0	0	0	0	0	0	0	0	0		0
0	0	0		1		0	0	0	0	0	1	0	0	0	0	0	0	0	0	0	0	0		0
0	0	0		1		0	0	0	0	0	1	0	0	0	0	0	0	0	0	0	0	0		0
0	1	0		2	1	1	0	0	0	0	0	0	0	0	0	0	0	0	0	1	0	0		0
0	0	0		1		0	0	0	0	0	1	0	0	0	0	0	0	0	0	0	0	0		0
0	0	0		1		0	0	0	0	0	1	0	0	0	0	0	0	0	0	0	0	0		0
0	0	1		1		0	0	0	0	0	1	0	0	0	0	0	0	0	0	0	0	0		0
0	0	0		1		0	0	0	0	0	1	0	0	0	0	0	0	0	0	0	0	0		0
0	0	0		1		0	0	0	0	0	1	0	0	0	0	0	0	0	0	0	0	0		0
0	0	0		1		0	0	0	0	0	1	0	0	0	0	0	0	0	0	0	0	0		0
1	0	0		1		0	0	0	0	0	0	1	0	0	0	0	0	0	0	0	0	0		0
0	0	0		1		0	0	0	0	0	1	0	0	0	0	0	0	0	0	0	0	0		0
0	0	0		1		0	0	0	0	0	1	0	0	0	0	0	0	0	0	0	0	0		0
0	0	0		1		0	0	0	0	0	1	0	0	0	0	0	0	0	0	0	0	0		0
0	0	0		1		0	0	0	0	0	1	0	0	0	0	0	0	0	0	0	0	0		0
0	0	0		2	1	1	0	0	0	0	0	0	0	1	0	0	0	0	0	0	0	0		0
0	0	0		1		0	0	0	0	0	1	0	0	0	0	0	0	0	0	0	0	0		0
0	0	0		1		0	0	0	0	0	1	0	0	0	0	0	0	0	0	0	0	0		0
0	0	0		1		0	0	0	0	0	1	0	0	0	0	0	0	0	0	0	0	0		0
0	0	1	ex amiga	2	2	0	0	1	0	0	0	0	0	1	0	0	0	0	0	0	0	0		0

sexual_5___2	sexual_5___3	sexual_5___4	sexual_5___5	sexual_5___6	sexual_5___88	sexual_6___1	sexual_6___2	sexual_6___3	sexual_6___4	sexual_6___5	sexual_6___6	sexual_6___7	sexual_6___88	sexual_6___89	sexual_6___90	otro_sex_6	papel	responsabilidades	decisiones	violencia	golpe	fuerte	avergonzados	insulto	embarazo	enojo	condon	mujer	pareja
0	0	0	0	1	0	0	0	0	0	0	0	0	0	0	0		3	2	3	3	3	2	3	3	3	3	1	1	1
0	0	0	0	1	0	0	0	0	0	0	0	0	0	0	0		3	3	3	3	3	3	3	1	1	3	1	1	1
0	0	0	1	0	0	0	0	0	0	0	1	0	0	0	0		1	3	2	3	3	1	2	1	2	3	1	1	1
0	0	0	0	0	1	0	0	0	0	0	0	0	0	0	0		3	3	3	3	3	3	3	2	3	3	1	1	1
0	0	0	0	0	1	0	0	0	0	0	0	0	0	0	0		2	1	2	2	2	2	2	3	3	3	3	1	2
0	0	0	0	0	1	0	0	0	0	0	0	0	0	0	0		2	2	2	1	1	1	1	1	1	1	1	1	1
0	0	0	0	0	1	0	0	0	0	0	0	0	0	0	0		3	1	3	3	3	2	2	2	2	2	2	2	1
0	0	0	0	1	0	0	0	0	0	0	0	0	0	0	0		3	3	2	3	3	1	1	1	3	3	1	1	1
0	0	0	0	0	1	0	0	0	0	0	0	0	0	0	0		1	3	3	3	3	3	3	3	3	1	3	3	1
0	0	0	0	1	0	0	0	0	0	0	0	0	0	0	0		1	1	1	3	3	3	3	3	1	3	1	3	3
0	0	0	0	1	0	0	0	0	0	0	0	0	0	0	0		3	1	3	3	3	3	3	1	3	1	1	1	1
0	0	0	0	1	0	0	0	0	0	0	0	0	0	0	0		3	1	3	3	3	3	1	1	3	1	1	1	1
0	0	0	0	1	0	0	0	0	0	0	0	0	0	0	0		1	1	3	3	3	3	2	2	3	3	1	1	1
0	0	0	0	1	0	0	0	0	0	0	0	0	0	0	0		3	1	3	3	3	3	3	1	3	1	1	1	1
0	0	0	0	1	0	0	0	0	0	0	0	0	0	0	0		3	3	3	3	3	1	3	3	3	3	1	1	1
0	0	0	0	1	0	0	0	0	0	0	0	0	0	0	0		3	3	3	3	3	1	2	1	3	3	1	1	1
0	0	0	0	1	0	0	0	0	0	0	0	0	0	0	0		1	1	3	3	3	2	3	1	1	3	3	3	1
0	0	0	0	0	1	0	0	0	0	0	0	0	0	0	0		3	3	3	3	3	2	3	1	3	3	1	1	1
0	0	0	0	1	0	0	0	0	0	0	0	0	0	0	0		1	1	3	3	3	3	3	3	1	3	1	1	1
0	0	0	0	0	1	0	0	0	0	0	0	0	0	0	0		1	1	3	3	3	1	3	1	3	1	1	1	1
0	0	0	0	1	0	0	0	0	0	0	0	0	0	0	0		3	3	3	3	3	3	3	2	1	2	1	1	1
0	0	0	0	1	0	0	0	0	0	0	0	0	0	0	0		3	1	3	3	3	3	3	3	3	3	1	3	1
0	0	0	0	0	0	0	0	0	0	0	0	0	1	0	0		3	3	3	3	3	2	3	3	3	3	1	1	1
0	0	0	0	1	0	0	0	0	0	0	0	0	0	0	0		3	3	3	3	3	3	3	1	3	2	1	1	1
0	0	0	0	1	0	0	0	0	0	0	0	0	0	0	0		1	3	3	3	3	3	3	3	3	3	1	1	1
0	0	0	0	1	0	0	0	0	0	0	0	0	0	0	0		1	1	2	1	3	1	1	3	3	2	2	3	2
0	0	0	0	0	1	0	0	0	0	0	0	0	0	0	0		1	1	3	3	3	3	3	1	3	1	1	1	3
0	0	0	0	1	0	0	0	0	0	0	0	0	0	0	0		3	3	3	3	3	3	1	2	3	3	1	1	1
0	0	0	0	1	0	0	0	0	0	0	0	0	0	0	0		1	1	1	3	3	3	2	2	3	2	1	1	2
0	0	0	0	0	1	0	0	0	0	0	0	0	0	0	0		1	1	2	3	3	1	3	1	1	1	1	1	1
0	0	0	0	1	0	0	0	0	0	0	0	0	0	0	0		3	3	3	3	3	3	3	1	3	3	1	1	1
0	0	0	0	1	0	0	0	0	0	0	0	0	0	0	0		1	1	1	3	3	3	3	1	2	3	3	1	1
0	0	0	0	1	0	0	0	0	0	0	0	0	0	0	0		2	1	2	2	2	3	3	2	3	3	1	1	1
0	0	0	0	1	0	0	0	0	0	0	0	0	0	0	0		1	1	1	1	3	1	1	3	1	2	2	1	2
0	0	0	0	1	0	0	0	0	0	0	0	0	0	0	0		3	3	3	3	3	3	3	3	3	3	1	1	1
0	0	0	0	1	0	0	0	0	0	0	0	0	0	0	0		1	2	1	3	2	3	1	3	1	3	3	1	1
0	0	0	0	1	0	0	0	0	0	0	0	0	0	0	0		3	3	2	3	3	3	3	1	3	3	1	1	1
0	0	0	0	1	0	0	0	0	0	0	0	0	0	0	0		3	1	3	3	3	3	3	3	1	3	1	1	2
0	0	0	0	1	0	0	0	0	0	0	0	0	0	0	0		2	1	3	3	3	1	1	3	3	3	1	1	3
0	0	0	0	1	0	0	0	0	0	0	0	0	0	0	0		1	1	2	3	3	1	1	1	1	3	1	1	1
0	0	0	0	1	0	0	0	0	0	0	0	0	0	0	0		3	1	3	3	3	3	3	1	1	1	1	1	3
0	0	0	0	1	0	0	0	0	0	0	0	0	0	0	0		3	2	3	3	3	1	2	3	1	2	2	1	2
0	0	0	0	1	0	0	0	0	0	0	0	0	0	0	0		1	3	3	3	3	2	3	3	2	3	1	1	1
0	0	0	0	1	0	0	0	0	0	0	0	0	0	0	0		1	1	1	3	3	3	3	3	1	1	1	1	1
0	0	0	0	1	0	0	0	0	0	0	0	0	0	0	0		1	1	3	3	3	3	3	3	1	3	1	1	1
0	0	0	0	1	0	0	0	0	0	0	0	0	0	0	0		1	1	3	3	3	1	2	1	1	3	1	1	1
0	0	0	0	1	0	0	0	0	0	0	0	0	0	0	0		2	3	3	3	2	1	3	3	3	3	1	1	1
0	0	0	0	1	0	0	0	0	0	0	0	0	0	0	0		2	1	1	3	3	2	2	1	1	1	1	1	1
0	0	0	0	1	0	0	0	0	0	0	0	0	0	0	0		3	1	3	3	3	1	1	3	3	1	1	1	2

ayuda_1	ayuda_2___1	ayuda_2___2	ayuda_2___3	ayuda_2___4	ayuda_2___5	ayuda_2___6	ayuda_2___7	ayuda_2___88	ayuda_2___89	ayuda_2___90	otro_ayuda_2	ayuda_3	ayuda_4___1	ayuda_4___2	ayuda_4___3	ayuda_4___4	ayuda_4___5	ayuda_4___6	ayuda_4___7	ayuda_4___8	ayuda_4___88	ayuda_4___89	ayuda_4___90	otro_ayuda_4	ayuda_5	ayuda_6
1	0	0	0	0	0	0	0	0	0	0		2	1	0	0	0	0	0	0	0	0	0	1	Psicologo/doctor	2	89
1	0	0	0	0	0	0	0	0	0	0		2	0	0	0	0	0	0	0	1	0	0	0		2	2
88	0	0	0	0	0	0	0	0	0	0		88	0	0	0	0	0	0	0	0	1	0	0		88	88
1	0	0	0	0	0	0	0	0	0	0		1	0	0	0	0	0	0	0	0	1	0	0		2	2
1	0	0	0	0	0	0	0	0	0	0		1	0	0	1	0	0	0	0	0	0	0	0		89	2
1	0	0	0	0	0	0	0	0	0	0		2	0	0	0	0	0	0	0	0	0	1	0		2	2
1	0	0	0	0	0	0	0	0	0	0		2	0	0	0	1	0	0	0	0	0	0	0		2	1
1	0	0	0	0	0	0	0	0	0	0		1	0	0	0	0	0	0	0	0	0	0	1	Mi tia	2	2
88	0	0	0	0	0	0	0	0	0	0		1	0	0	0	0	0	0	0	0	1	0	0		2	1
1	0	0	0	0	0	0	0	0	0	0		1	0	0	1	0	0	0	0	0	0	0	0		89	1
1	0	0	0	0	0	0	0	0	0	0		88	0	0	1	0	0	0	0	0	0	0	0		2	1
1	0	0	0	0	0	0	0	0	0	0		1	0	0	0	0	0	0	0	0	1	0	0		89	2
1	0	0	0	0	0	0	0	0	0	0		1	0	0	0	0	0	0	0	0	1	0	0		2	2
1	0	0	0	0	0	0	0	0	0	0		88	0	0	1	0	0	0	0	0	0	0	0		2	1
1	0	0	0	0	0	0	0	0	0	0		1	0	0	0	0	0	0	0	0	1	0	0		2	1
2	0	1	0	0	0	0	0	0	0	0		2	0	0	1	1	0	0	0	0	0	0	0		1	2
1	0	0	0	0	0	0	0	0	0	0		1	0	0	1	0	0	0	0	0	0	0	0		2	2
1	0	0	0	0	0	0	0	0	0	0		2	0	0	0	0	0	0	0	1	0	0	0		2	2
1	0	0	0	0	0	0	0	0	0	0		2	0	0	0	1	0	0	0	0	0	0	0		2	2
1	0	0	0	0	0	0	0	0	0	0		2	1	0	0	0	0	0	0	1	0	0	0		2	2
1	0	0	0	0	0	0	0	0	0	0		2	1	0	1	0	1	0	0	1	0	0	0		2	2
1	0	0	0	0	0	0	0	0	0	0		2	0	0	0	0	0	0	0	0	0	0	1		2	2
88	0	0	0	0	0	0	0	0	0	0		1	0	0	0	0	0	0	0	0	0	1	0		2	89
1	0	0	0	0	0	0	0	0	0	0		88	0	0	0	0	0	0	0	0	1	0	0		88	88
1	0	0	0	0	0	0	0	0	0	0		1	0	0	0	0	0	0	0	0	0	0	1	major amiga	2	1
1	0	0	0	0	0	0	0	0	0	0		2	0	0	0	0	0	0	1	0	0	0	0		89	1
2	0	0	0	0	0	1	0	0	0	0		2	0	0	1	0	0	0	0	0	0	0	0		2	2
1	0	0	0	0	0	0	0	0	0	0		2	0	0	0	0	0	0	0	0	1	0	0		2	2
1	0	0	0	0	0	0	0	0	0	0		1	0	0	1	0	0	0	0	0	0	0	0		2	1
2	0	0	0	1	0	0	0	0	0	0		1	0	0	0	1	0	0	0	0	0	0	0		2	1
1	0	0	0	0	0	0	0	0	0	0		1	0	0	0	1	0	0	0	0	0	0	0		89	1
1	0	0	0	0	0	0	0	0	0	0		2	0	0	0	0	0	1	0	0	0	0	0		1	2
2	0	0	0	0	0	0	0	0	1	0		1	0	0	0	0	0	0	0	1	0	0	0		89	2
1	0	0	0	0	0	0	0	0	0	0		1	0	0	0	0	1	0	0	1	0	0	0		1	1
2	1	0	0	0	0	0	0	0	0	0		2	0	0	0	0	0	0	0	1	0	0	0		2	2
2	0	0	0	0	1	0	0	0	0	1	atracadores	2	0	0	0	0	0	0	0	0	0	0	1		1	2
1	0	0	0	0	0	0	0	0	0	0		2	0	0	1	0	1	0	1	1	0	0	0		2	2
1	0	0	0	0	0	0	0	0	0	0		2	0	0	0	0	0	0	0	0	1	0	0		88	88
1	0	0	0	0	0	0	0	0	0	0		1	0	0	1	0	0	0	0	0	0	0	0		2	2
1	0	0	0	0	0	0	0	0	0	0		1	0	0	1	0	0	0	0	0	0	0	0		2	1
1	0	0	0	0	0	0	0	0	0	0		1	0	0	1	0	0	0	0	0	0	0	0		2	1
1	0	0	0	0	0	0	0	0	0	0		1	0	0	1	1	0	0	0	0	0	0	0		89	1
1	0	0	0	0	0	0	0	0	0	0		1	0	0	0	1	0	0	0	0	0	0	0		2	2
1	0	0	0	0	0	0	0	0	0	0		2	0	0	0	1	0	0	0	0	0	0	0		2	2
2	0	1	0	0	0	0	0	0	0	0		2	1	0	0	1	0	0	0	0	0	0	0		2	1
2	0	0	0	0	0	0	0	0	0	1	madre	2	0	0	1	0	0	0	0	0	0	0	0		2	2
2	0	1	0	0	0	0	0	0	0	0		1	0	0	1	0	0	0	0	0	0	0	0		2	2
2	0	1	0	0	0	0	0	0	0	0		1	0	0	1	0	0	0	1	0	0	0	0		2	89
2	0	1	0	0	0	0	0	0	0	0		2	0	0	1	1	0	0	0	0	0	0	0		2	2

ayuda_7	ayuda_8___1	ayuda_8___2	ayuda_8___3	ayuda_8___4	ayuda_8___5	ayuda_8___6	ayuda_8___7	ayuda_8___8	ayuda_8___88	ayuda_8___89	ayuda_8___90	otro_ayuda_8	ayuda_9	form_1_complete
2	0	0	0	0	0	0	0	1	0	0	0		2	2
2	0	0	0	0	0	0	0	1	0	0	0		2	2
88	0	0	0	0	0	0	0	0	0	0	0			2
1	0	0	0	0	0	0	0	0	0	0	0			2
1	0	0	0	0	0	0	0	0	0	0	0			2
2	0	0	0	0	0	0	0	1	0	0	0		2	2
1	0	0	0	0	0	0	0	0	0	0	0			2
1	0	0	0	0	0	0	0	0	0	0	0			2
88	0	0	0	0	0	0	0	0	0	0	0			2
1	0	0	0	0	0	0	0	0	0	0	0			2
1	0	0	0	0	0	0	0	0	0	0	0			2
1	0	0	0	0	0	0	0	0	0	0	0			2
1	0	0	0	0	0	0	0	0	0	0	0			2
1	0	0	0	0	0	0	0	0	0	0	0			2
1	0	0	0	0	0	0	0	0	0	0	0			2
1	0	0	0	0	0	0	0	0	0	0	0			2
1	0	0	0	0	0	0	0	0	0	0	0			2
2	0	0	0	1	0	0	0	1	0	0	0		1	2
1	0	0	0	0	0	0	0	0	0	0	0			2
2	0	0	0	0	0	0	0	0	1	0	0		88	2
1	0	0	0	0	0	0	0	0	0	0	0			2
1	0	0	0	0	0	0	0	0	0	0	0			2
2	0	0	0	0	0	0	0	1	0	0	0		1	2
88	0	0	0	0	0	0	0	0	0	0	0			2
2	0	0	0	0	0	0	0	1	0	0	0		2	2
1	0	0	0	0	0	0	0	0	0	0	0			2
1	0	0	0	0	0	0	0	0	0	0	0			2
1	0	0	0	0	0	0	0	0	0	0	0			2
2	0	0	0	0	0	0	0	1	0	0	0		1	2
2	0	0	0	1	0	1	0	0	0	0	0		1	2
1	0	0	0	0	0	0	0	0	0	0	0			2
1	0	0	0	0	0	0	0	0	0	0	0			2
2	0	0	0	0	0	0	0	1	0	0	0		2	2
1	0	0	0	0	0	0	0	0	0	0	0			2
2	0	0	0	0	0	0	0	1	0	0	0		1	2
2	0	0	0	0	0	0	0	0	0	0	1		2	2
2	0	0	0	1	0	1	0	0	0	0	0		2	2
2	0	0	0	0	0	0	0	0	1	0	0		2	2
2	0	0	0	1	0	0	0	0	0	0	0		2	2
1	0	0	0	0	0	0	0	0	0	0	0			2
1	0	0	0	0	0	0	0	0	0	0	0			2
1	0	0	0	0	0	0	0	0	0	0	0			2
1	0	0	0	0	0	0	0	0	0	0	0			2
2	1	0	0	0	0	0	0	0	0	0	0		2	2
1	0	0	0	0	0	0	0	0	0	0	0			2
1	0	0	0	0	0	0	0	0	0	0	0			2
88	0	0	0	0	0	0	0	0	0	0	0			2
2	0	0	0	0	0	1	0	0	0	0	0		2	2
2	0	0	0	0	0	0	0	1	0	0	0		2	2
