# Supplementary material for: Thoughts of self-harm in adolescents: Relationship with violence in the Dominican Republic
Source: PLOS Glob Public Health. 2024 Jan 8;4(1):e0002711. doi: 10.1371/journal.pgph.0002711 (PMC10773958; doi:10.1371/journal.pgph.0002711)
Supplement: S2 Dataset — (PDF) [file pgph.0002711.s004.pdf]

# Alcance y tipos de violencia en adolescentes PID 34

Libro de códigos

## Libro de códigos del diccionario de datos

11-16-2022 14:53

Collapse all instruments

| | # | variable/nombre del campo | Etiqueta de Campo<br>*Nota de campo* | Atributos de campo (tipo de campo, validación, opciones, una lógica ramificada, cálculos, etc.) |
|---|---|---|---|---|
| Instrumento: **Form 1** (form_1) Enabled as survey Collapse | | | | |
| | 1 | record_id | Record ID | text |
| | 2 | rural | Section Header: *Muchas gracias por acceder a ser parte de este estudio. Voy a hacerte un grupo de preguntas acerca de ti, y luego hablaremos de tus experiencias y percepciones acerca de violencia. Algunas de estas preguntas pueden ser difíciles, personales o incómodas de responder. Debido a esto, te quiero aclarar que tus respuestas se mantendrán estrictamente confidenciales, y si alguna pregunta te hace sentir incómoda, puedes elegir no responder, o también puedes parar la encuesta en cualquier momento. ¿Tienes alguna pregunta antes de comenzar?*<br>Donde vives? | radio, Required<br>0 Batey<br>1 La Romana |
| | 3 | edad | ¿Cuántos años tienes? | text (number), Required |
| | 4 | sexo | ¿Cuál es tu sexo? | radio, Required<br>1 Masculino<br>2 Femenino<br>3 Transgénero/Transexual<br>4 Otro<br>88 Rehúsa responder<br>89 No sabe |
| | 5 | sexo_otro<br>Mostrar el archivo SÓLO si: [sexo] = '4' | Si es otro, ¿Cuál? | text |
| | 6 | nacion | ¿Dónde naciste? | radio, Required<br>1 República Dominicana<br>2 Haití<br>3 Puerto rico<br>4 EEUU<br>5 Otro<br>88 Rehúsa responder<br>89 No sabe |
| | 7 | nac_otro<br>Mostrar el archivo SÓLO si: [nacion] = '5' | Si es otro, ¿Cuál? | text |
| | 8 | curso | ¿Hasta cuál curso llegaste? | radio, Required<br>1 Primaria, incompleta<br>2 Primaria, completa<br>3 Bachillerato, incompleta<br>4 Bachillerato, completa<br>5 Universidad, incompleta<br>6 Universidad, completa<br>88 Rehúsa responder<br>89 No sabe |

| | | | | |
|---|---|---|---|---|
| | 9 | asistencia | Antes de salir de vacaciones, ¿Has asistido a la escuela? | radio, Required<br>1 Sí<br>2 No<br>88 Rehúsa responder<br>89 No sabe |
| | 10 | abandono | ¿Alguna vez has abandonado la escuela? | radio, Required<br>1 Sí<br>2 No<br>88 Rehúsa responder<br>89 No sabe |
| | 11 | anos_ab<br>Mostrar el archivo SÓLO si: [abandono] = '1' | ¿Cuántos años tenías la primera vez que abandonaste la escuela? | text (number) |
| | 12 | razon_ab<br>Mostrar el archivo SÓLO si: [abandono] = '1' | ¿Por qué abandonaste la escuela? Elija todas las que apliquen | checkbox<br>1 razon_ab___1 Tenía que trabajar/ganar dinero<br>2 razon_ab___2 No me gusta estudiar<br>3 razon_ab___3 La escuela estaba muy lejos<br>4 razon_ab___4 Los compañeros/profesores me molestaban<br>5 razon_ab___5 Otra razón<br>88 razon_ab___88 Rehúsa responder<br>89 razon_ab___89 No sabe |
| | 13 | otro_razon_ab<br>Mostrar el archivo SÓLO si: [razon_ab(5)] = '1' | Si es otra, ¿Cuál? | text |
| | 14 | vive | ¿Con quién vives? Elija todas las que apliquen | checkbox, Required<br>1 vive___1 Mi familia (ambos padres, hermanos...)<br>2 vive___2 Sólo uno de mis padres<br>3 vive___3 Familiares cercanos (abuela, hermanos...)<br>4 vive___4 Otros familiares (tío, padrino, etc..)<br>5 vive___5 Amigos<br>6 vive___6 Otro<br>88 vive___88 Rehúsa responder<br>89 vive___89 No sabe |
| | 15 | otro_vive<br>Mostrar el archivo SÓLO si: [vive(6)] = '1' | Si es otro, ¿Cuál? | text |
| | 16 | trabajo | ¿Actualmente trabajas? | radio, Required<br>1 Sí<br>2 No<br>88 Rehúsa responder<br>89 No sabe |

| | | | | |
|---|---|---|---|---|
| | 17 | tipo_trabajo<br>Mostrar el archivo SÓLO si:<br>[trabajo] = '1' | Si trabajas, ¿Qué tipo de trabajo realizas? | radio<br>1 \| Trabajo formal, documentado (en algún negocio con beneficios de ley)<br>2 \| Trabajo informal, indocumentado (trabajo sin beneficio, ej: delivery de colmado)<br>3 \| Trabajo independiente (vendedor ambulante, negocio propio)<br>4 \| Otro<br>88 \| Rehúsa responder |
| | 18 | otro_tipo<br>Mostrar el archivo SÓLO si:<br>[tipo_trabajo] = '4' | Si es otro, ¿Cuál? | text |
| | 19 | fisica_1 | Section Header: *Violencia Física Ahora te voy a hacer algunas preguntas acerca de violencia. Pero primero, me gustaría aclarar que la Violencia Física es cualquier acto intencional usando fuerza física para causar lesiones internas o externas. ¿Tienes alguna pregunta acerca de este término?*<br>¿Alguna ves has sido víctima de violencia física? | radio, Required<br>1 \| No<br>2 \| Sí<br>88 \| Rehúsa responder<br>89 \| No sabe |
| | 20 | fisica_2<br>Mostrar el archivo SÓLO si:<br>[fisica_1] = '2' | ¿Has sido víctima de violencia física en los últimos 60 días? | radio<br>1 \| No<br>2 \| Sí<br>88 \| Rehúsa responder<br>89 \| No sabe |
| | 21 | fisica_3 | Alguna vez: (Elija todas las opciones que apliquen) | checkbox<br>1 \| fisica_3___1 \| Has sido abofeteado, te han pegado un puñetazo o pateado<br>2 \| fisica_3___2 \| Has sido víctima de intento de secuestro<br>3 \| fisica_3___3 \| Has sido apuñalado<br>4 \| fisica_3___4 \| Has sido atado o asfixiado<br>5 \| fisica_3___5 \| Has sido amenazado con un arma<br>6 \| fisica_3___6 \| Ninguna de las anteriores<br>88 \| fisica_3___88 \| Rehúsa responder |
| | 22 | fisica_4<br>Mostrar el archivo SÓLO si:<br>[fisica_3(1)] = '1' or [fisica_3(2)] = '1' or [fisica_3(3)] = '1' or [fisica_3(4)] = '1' or [fisica_3(5)] = '1' | ¿Quién cometió este acto de violencia? Elija todas las que aplican | checkbox<br>1 \| fisica_4___1 \| Pareja actual<br>2 \| fisica_4___2 \| Ex-pareja<br>3 \| fisica_4___3 \| Padre<br>4 \| fisica_4___4 \| Otro familiar o guardián<br>5 \| fisica_4___5 \| Persona de autoridad (oficial de policía, CESTUR, etc.)<br>6 \| fisica_4___6 \| Compañero de clases<br>7 \| fisica_4___7 \| Profesor<br>88 \| fisica_4___88 \| Rehúsa responder<br>89 \| fisica_4___89 \| No sabe/Persona desconocida<br>90 \| fisica_4___90 \| Otra persona |
| | 23 | otro_fisica_4<br>Mostrar el archivo SÓLO si:<br>[fisica_4(90)] = '1' | Si es otra, ¿Cuál? | text |

| | | | | |
|---|---|---|---|---|
| | 24 | fisica_5 | Alguna vez has: (Elija todas las opciones que apliquen) | checkbox, Required<br>1 \| fisica_5___1 \| Abofeteado, pegado un puñetazo o pateado a otra persona?<br>2 \| fisica_5___2 \| Intentado secuestrar a alguien?<br>3 \| fisica_5___3 \| Apuñalado a alguien?<br>4 \| fisica_5___4 \| Atado o asfixiado a otra persona?<br>5 \| fisica_5___5 \| Amenazado a alguien con un arma?<br>6 \| fisica_5___6 \| Ninguna de las anteriores<br>88 \| fisica_5___88 \| Rehúsa responder |
| | 25 | fisica_6<br>Mostrar el archivo SÓLO si: [fisica_5(1)] = '1' or [fisica_5(2)] = '1' or [fisica_5(3)] = '1' or [fisica_5(4)] = '1' or [fisica_5(5)] = '1' | ¿A quién le hiciste esto? Elija todas las que aplican | checkbox<br>1 \| fisica_6___1 \| Pareja actual<br>2 \| fisica_6___2 \| Ex-pareja romántica/sexual<br>3 \| fisica_6___3 \| Padre<br>4 \| fisica_6___4 \| Otro familiar o guardián<br>5 \| fisica_6___5 \| Persona de autoridad (oficial de policía, CESTUR, etc.)<br>6 \| fisica_6___6 \| Compañero de clases<br>7 \| fisica_6___7 \| Profesor<br>88 \| fisica_6___88 \| Rehúsa responder<br>89 \| fisica_6___89 \| No sabe/Persona desconocida<br>90 \| fisica_6___90 \| Otra persona |
| | 26 | otro_fisica_6<br>Mostrar el archivo SÓLO si: [fisica_6(90)] = '1' | Si es otra, ¿Cuál? | text |
| | 27 | emocional_1 | Section Header: *Violencia Emocional y/o Psicológica Gracias por tu tiempo. Ahora voy a hacer algunas preguntas acerca de violencia emocional o psicológica. Recuerda que la violencia emocional o psicológica quiere decir cualquier acción que te hace sentir mal, humillado o disminuye tu autoestima sin utilizar fuerza física. ¿Tienes alguna pregunta, antes de iniciar?*<br>¿Alguna vez has sido víctima de violencia emocional o psicológica? | radio, Required<br>1 \| No<br>2 \| Sí<br>88 \| Rehúsa responder<br>89 \| No sabe |
| | 28 | emocional_2<br>Mostrar el archivo SÓLO si: [emocional_1] = '2' | Durante los últimos 60 días, ¿has sido víctima de violencia emocional o psicológica? | radio<br>1 \| No<br>2 \| Sí<br>88 \| Rehúsa responder<br>89 \| No sabe |
| | 29 | emocional_3 | Alguna vez: (Elija todas las opciones que apliquen) | checkbox<br>1 \| emocional_3___1 \| Has sido insultado, o le han hecho sentir mal<br>2 \| emocional_3___2 \| Has sido humillado o minimizado enfrente de otras personas<br>3 \| emocional_3___3 \| Has sido intimidado o asustado por alguien<br>4 \| emocional_3___4 \| Has sido amenazado o alguien amenazó a un ser querido<br>5 \| emocional_3___5 \| Ninguna de las anteriores<br>88 \| emocional_3___88 \| Rehúsa responder |

| | # | Variable / Field Name | Field Label | Field Attributes |
|---|---|---|---|---|
| | 30 | emocional_4<br>Mostrar el archivo SÓLO si: [emocional_3(1)] = '1' or [emocional_3(2)] = '1' or [emocional_3(3)] = '1' or [emocional_3(4)] = '1' | ¿Quién te hizo sentir así? Elija todas las que aplican | checkbox<br>1 \| emocional_4___1 \| Pareja actual<br>2 \| emocional_4___2 \| Ex-pareja<br>3 \| emocional_4___3 \| Padre<br>4 \| emocional_4___4 \| Otro familiar o guardián<br>5 \| emocional_4___5 \| Persona de autoridad (oficial de policía, CESTUR, etc.)<br>6 \| emocional_4___6 \| Compañero de clases<br>7 \| emocional_4___7 \| Profesor<br>88 \| emocional_4___88 \| Rehúsa responder<br>89 \| emocional_4___89 \| No sabe/Persona desconocida<br>90 \| emocional_4___90 \| Otra persona |
| | 31 | otro_emoc_4<br>Mostrar el archivo SÓLO si: [emocional_4(90)] = '1' | Si es otra, ¿Cuál? | text |
| | 32 | emocional_5 | Alguna vez has: (Elija todas las opciones que apliquen) | checkbox, Required<br>1 \| emocional_5___1 \| Insultado, o has hecho que otra persona se sienta mal?<br>2 \| emocional_5___2 \| Humillado o minimizado a otras personas enfrente de los demás?<br>3 \| emocional_5___3 \| Intimidado o asustado a alguien<br>4 \| emocional_5___4 \| Amenazado a alguien, o amenazaste a un ser querido de otra persona?<br>5 \| emocional_5___5 \| Ninguna de las anteriores<br>88 \| emocional_5___88 \| Rehúsa responder |
| | 33 | emocional_6<br>Mostrar el archivo SÓLO si: [emocional_5(1)] = '1' or [emocional_5(2)] = '1' or [emocional_5(3)] = '1' or [emocional_5(4)] = '1' | ¿A quién le hiciste esto? Elija todas las que aplican | checkbox<br>1 \| emocional_6___1 \| Pareja actual<br>2 \| emocional_6___2 \| Ex-pareja<br>3 \| emocional_6___3 \| Padre<br>4 \| emocional_6___4 \| Otro familiar o guardián<br>5 \| emocional_6___5 \| Persona de autoridad (oficial de policía, CESTUR, etc.)<br>6 \| emocional_6___6 \| Compañero de clases<br>7 \| emocional_6___7 \| Profesor<br>88 \| emocional_6___88 \| Rehúsa responder<br>89 \| emocional_6___89 \| No sabe/Persona desconocida<br>90 \| emocional_6___90 \| Otra persona |
| | 34 | otro_emoc_6<br>Mostrar el archivo SÓLO si: [emocional_6(90)] = '1' | Si es otra, ¿Cuál? | text |
| | 35 | sexual_1 | Section Header: *Violencia Sexual Gracias nueva vez, por tu paciencia. Ahora hablaremos de Violencia Sexual. La violencia sexual significa cualquier tipo de contacto sexual entre dos o más personas, donde una de las personas no haya dado su permiso, o no este de acuerdo con lo que está pasando en ese momento. ¿Tienes alguna pregunta o duda sobre este término?*<br>¿Alguna vez has sido víctima de violencia sexual? | radio, Required<br>1 \| No<br>2 \| Sí<br>88 \| Rehúsa responder<br>89 \| No sabe |

| | | | | |
|---|---|---|---|---|
| | 36 | sexual_2<br><br>Mostrar el archivo SÓLO si:<br>[sexual_1] = '2' | Durante los últimos 60 días, ¿has sido víctima de violencia sexual? | radio<br>1 No<br>2 Sí<br>88 Rehúsa responder<br>89 No sabe |
| | 37 | sexual_3 | Alguna ves te ha pasado que: (Elija todas las que apliquen) | checkbox<br>1 sexual_3___1 Has sido víctima de alguien intentando introducir su pene u otro objeto por vía vaginal o anal<br>2 sexual_3___2 Has sido obligado/a a realizar sexo oral a otra persona<br>3 sexual_3___3 Has sido penetrado/a forzosamente por vía vaginal o anal<br>4 sexual_3___4 Has sido obligado/a a tocar o besar a otra persona en contra de tu voluntad<br>5 sexual_3___5 Has sido tocado/a ó besado/a en contra de tu voluntad<br>6 sexual_3___6 Ninguna de las anteriores<br>88 sexual_3___88 Rehúsa responder |
| | 38 | sexual_4<br><br>Mostrar el archivo SÓLO si:<br>[sexual_3(1)] = '1' or [sexual_3(2)] = '1' or [sexual_3(3)] = '1' or [sexual_3(4)] = '1' or [sexual_3(5)] = '1' | ¿Quién te hizo esto? (Elija todas las opciones que apliquen) | checkbox<br>1 sexual_4___1 Pareja actual<br>2 sexual_4___2 Ex-pareja<br>3 sexual_4___3 Padre<br>4 sexual_4___4 Otro familiar o guardián<br>5 sexual_4___5 Persona de autoridad (oficial de policía, CESTUR, etc.)<br>6 sexual_4___6 Compañero de clases<br>7 sexual_4___7 Profesor<br>88 sexual_4___88 Rehúsa responder<br>89 sexual_4___89 No sabe/Persona desconocida<br>90 sexual_4___90 Otra persona |
| | 39 | otro_sex_4<br><br>Mostrar el archivo SÓLO si:<br>[sexual_4(90)] = '1' | Si es otro, ¿Cuál? | text |
| | 40 | sexual_5 | Alguna ves has: (Elija todas las que apliquen) | checkbox, Required<br>1 sexual_5___1 Intentando forzar tu pene u otro objeto en la vaginal o ano de otra persona?<br>2 sexual_5___2 Obligado a alguien a realizarte sexo oral?<br>3 sexual_5___3 Introducido tu pene u objeto en la vaginal o ano de otra persona en contra de su voluntad?<br>4 sexual_5___4 Obligado a alguien a tocarte o besar en contra de tu voluntad?<br>5 sexual_5___5 Tocado ó besado en contra de su voluntad?<br>6 sexual_5___6 Ninguna de las anteriores<br>88 sexual_5___88 Rehúsa responder |

| | | | | |
|---|---|---|---|---|
| | 41 | sexual_6<br><br>Mostrar el archivo SÓLO si: [sexual_5(1)] = '1' or [sexual_5(2)] = '1' or [sexual_5(3)] = '1' or [sexual_5(4)] = '1' or [sexual_5(5)] = '1' | ¿A quién le hiciste esto? (Elija las opciones que apliquen) | checkbox<br>1 sexual_6___1 Pareja actual<br>2 sexual_6___2 Ex-pareja<br>3 sexual_6___3 Padre<br>4 sexual_6___4 Otro familiar o guardián<br>5 sexual_6___5 Persona de autoridad (oficial de policía, CESTUR, etc.)<br>6 sexual_6___6 Compañero de clases<br>7 sexual_6___7 Profesor<br>88 sexual_6___88 Rehúsa responder<br>89 sexual_6___89 No sabe/Persona desconocida<br>90 sexual_6___90 Otra persona |
| | 42 | otro_sex_6<br><br>Mostrar el archivo SÓLO si: [sexual_6(90)] = '1' | Si es otro, ¿Cuál? | text |
| | 43 | papel | Section Header: *Gracias por tu paciencia con estas preguntas. Ahora te voy a leer algunas oraciones. Por favor dime si estas de acuerdo, si estas en desacuerdo, o si no estas seguro acerca de las ideas que te voy a mencionar. Quiero recordarte que toda esta información es completamente confidencial, y puedes rehusar responder cualquier pregunta o parar en cualquier momento.*<br><br>El papel mas importante de una mujer es cuidar la casa y cocinar | radio (Matriz), Required<br>1 De acuerdo<br>2 No estoy seguro<br>3 En desacuerdo |
| | 44 | responsabilidades | Cambiar pañales, bañar y alimentar a los niños son responsabilidades de la madre | radio (Matriz), Required<br>1 De acuerdo<br>2 No estoy seguro<br>3 En desacuerdo |
| | 45 | decisiones | Un hombre debe tener la última palabra acerca de decisiones en la casa | radio (Matriz), Required<br>1 De acuerdo<br>2 No estoy seguro<br>3 En desacuerdo |
| | 46 | violencia | Una mujer debe tolerar momentos de violencia para mantener la familia junta | radio (Matriz), Required<br>1 De acuerdo<br>2 No estoy seguro<br>3 En desacuerdo |
| | 47 | golpe | Hay momentos cuando una mujer merece ser golpeada | radio (Matriz), Required<br>1 De acuerdo<br>2 No estoy seguro<br>3 En desacuerdo |
| | 48 | fuerte | Para ser un hombre, hay que ser fuerte | radio (Matriz), Required<br>1 De acuerdo<br>2 No estoy seguro<br>3 En desacuerdo |
| | 49 | avergonzados | Los hombres deberían estar avergonzados si no pueden tener una erección | radio (Matriz), Required<br>1 De acuerdo<br>2 No estoy seguro<br>3 En desacuerdo |
| | 50 | insulto | Si alguien me insulta, defenderé mi reputación, con fuerza de ser necesario | radio (Matriz), Required<br>1 De acuerdo<br>2 No estoy seguro<br>3 En desacuerdo |

| | | | | |
|---|---|---|---|---|
| | 51 | embarazo | Es responsabilidad de la mujer evitar quedar embarazada | radio (Matriz), Required<br>1 De acuerdo<br>2 No estoy seguro<br>3 En desacuerdo |
| | 52 | enojo | Me enojaría si mi pareja me pidiera ponerme un condón | radio (Matriz), Required<br>1 De acuerdo<br>2 No estoy seguro<br>3 En desacuerdo |
| | 53 | condon | Tanto un hombre como la mujer pueden sugerir usar un condón | radio (Matriz), Required<br>1 De acuerdo<br>2 No estoy seguro<br>3 En desacuerdo |
| | 54 | mujer | Si una mujer queda embarazada, el niño es responsabilidad de ambos | radio (Matriz), Required<br>1 De acuerdo<br>2 No estoy seguro<br>3 En desacuerdo |
| | 55 | pareja | Un hombre/mujer debería saber que le gusta a su pareja durante el sexo | radio (Matriz), Required<br>1 De acuerdo<br>2 No estoy seguro<br>3 En desacuerdo |
| | 56 | ayuda_1 | Section Header: *Buscar ayuda*<br>Actualmente, ¿te sientes expuesto o vulnerable a cualquier tipo de violencia? | radio, Required<br>1 No<br>2 Sí<br>88 Rehúsa responder<br>89 No sabe |
| | 57 | ayuda_2<br>Mostrar el archivo SÓLO si:<br>[ayuda_1] = '2' | ¿Quién te hace sentirte inseguro? Elija todas las que aplican | checkbox<br>1 ayuda_2___1 Pareja actual<br>2 ayuda_2___2 Ex-pareja<br>3 ayuda_2___3 Padre<br>4 ayuda_2___4 Otro familiar o guardián<br>5 ayuda_2___5 Persona de autoridad (oficial de policía, CESTUR, etc.)<br>6 ayuda_2___6 Compañero de clases<br>7 ayuda_2___7 Profesor<br>88 ayuda_2___88 Rehúsa responder<br>89 ayuda_2___89 No sabe/Persona desconocida<br>90 ayuda_2___90 Otra persona |
| | 58 | otro_ayuda_2<br>Mostrar el archivo SÓLO si:<br>[ayuda_2(90)] = '1' | Si es otra, ¿Cuál? | text |
| | 59 | ayuda_3 | ¿Alguna vez has hablado con alguien de confianza acerca de alguno de los eventos de violencia que hemos hablado el día de hoy? | radio<br>1 No<br>2 Sí<br>88 Rehúsa responder<br>89 No sabe |

| | | | | |
|---|---|---|---|---|
| | 60 | ayuda_4 | ¿A quién estaría dispuesto a hablarle acerca de sus experiencias de violencia? Elija todas las que aplican | checkbox, Required<br>1 \| ayuda_4___1 \| Pareja actual<br>2 \| ayuda_4___2 \| Ex-pareja<br>3 \| ayuda_4___3 \| Padre<br>4 \| ayuda_4___4 \| Otro familiar o guardián<br>5 \| ayuda_4___5 \| Persona de autoridad (oficial de policía, CESTUR, etc.)<br>6 \| ayuda_4___6 \| Compañero de clases<br>7 \| ayuda_4___7 \| Profesor<br>8 \| ayuda_4___8 \| Psicólogo o doctor<br>88 \| ayuda_4___88 \| Rehúsa responder<br>89 \| ayuda_4___89 \| No sabe/Persona desconocida<br>90 \| ayuda_4___90 \| Otra persona |
| | 61 | otro_ayuda_4<br>Mostrar el archivo SÓLO si: [ayuda_4(90)] = '1' | Si es otra, ¿Cuál? | text |
| | 62 | ayuda_5 | En caso de ser una víctima o perpetrador de violencia, ¿estarías interesado en recibir servicios de consejería y salud mental en la Clínica de Familia? | radio, Required<br>1 \| No<br>2 \| Sí<br>88 \| Rehúsa responder<br>89 \| No sabe |
| | 63 | ayuda_6 | ¿Sabes dónde puedes ir en caso de ser una víctima de violencia física, emocional o sexual? | radio, Required<br>1 \| No<br>2 \| Sí<br>88 \| Rehúsa responder<br>89 \| No sabe |
| | 64 | ayuda_7 | ¿Alguna vez has pensado en hacerte daño a usted mismo o alguna otra persona? | radio, Required<br>1 \| No<br>2 \| Sí<br>88 \| Rehúsa responder<br>89 \| No sabe |
| | 65 | ayuda_8<br>Mostrar el archivo SÓLO si: [ayuda_7] = '2' | ¿A quién has pensado hacerle daño? | checkbox<br>1 \| ayuda_8___1 \| Pareja actual<br>2 \| ayuda_8___2 \| Ex-pareja<br>3 \| ayuda_8___3 \| Padre<br>4 \| ayuda_8___4 \| Otro familiar o guardián<br>5 \| ayuda_8___5 \| Persona de autoridad (oficial de policía, CESTUR, etc.)<br>6 \| ayuda_8___6 \| Compañero de clases<br>7 \| ayuda_8___7 \| Profesor<br>8 \| ayuda_8___8 \| A mí mismo/a<br>88 \| ayuda_8___88 \| Rehúsa responder<br>89 \| ayuda_8___89 \| No sabe/Persona desconocida<br>90 \| ayuda_8___90 \| Otra persona |
| | 66 | otro_ayuda_8<br>Mostrar el archivo SÓLO si: [ayuda_8(90)] = '1' | Si es otra, ¿Cuál? | text |

| | | | | |
|---|---|---|---|---|
| | 67 | ayuda_9<br><br>Mostrar el archivo SÓLO si:<br>[ayuda_7] = '2' | ¿Alguna vez has pensado en buscar ayuda acerca de estos pensamientos? | radio<br>1 No<br>2 Sí<br>88 Rehúsa responder<br>89 No sabe |
| | 68 | form_1_complete | Section Header: *Form Status*<br><br>Complete? | dropdown<br>0 Incomplete<br>1 Unverified<br>2 Complete |
