# Supplementary material for: Thoughts of self-harm in adolescents: Relationship with violence in the Dominican Republic
Source: PLOS Glob Public Health. 2024 Jan 8;4(1):e0002711. doi: 10.1371/journal.pgph.0002711 (PMC10773958; doi:10.1371/journal.pgph.0002711)
Supplement: Attachment — Submitted filename: PGPH-D-23-00846 marked.pdf [file pgph.0002711.s005.pdf]

// PLOS Global Public Health

Thoughts of self-harm in adolescents: relationship with violence in the Dominican Republic

--Manuscript Draft--

**Manuscript Number:**	PGPH-D-23-00846
**Article Type:**	Research Article
**Full Title:**	Thoughts of self-harm in adolescents: relationship with violence in the Dominican Republic
**Short Title:**	Self harm thoughts in Dominican adolescents
**Corresponding Author:**	Kelsey Badger<br>Columbia University Columbia College<br>New York, NY UNITED STATES
**Order of Authors:**	Kelsey Badger
	Pamela Antonia Baez Caraballo, M.D MSc
	Ahzyris Gibbs
	Luz Messina
	Mina Halpern
	Silvia Amesty
**Keywords:**	Adolescents; self harm thoughts; suicidality; Dominican Republic; intimate partner violence; Violence
**Abstract:**	Violence against adolescents is a pressing health problem with long-term implications for future physical and mental well-being. One indication of poor mental well-being of adolescents is thoughts of self-harm, which are linked to suicidal ideation and completion. Although previous research has associated experiences of violence in childhood and adolescence with self-harm thoughts and behaviors, much of the research has been conducted only in high-income countries. This study aimed to examine the correlation between violence against adolescents and self-harm thoughts in the Dominican Republic (DR). Cross-sectional survey data was collected at a community-based clinic from participants aged 13-20. Participants were recruited through the clinic’s adolescent program, and verbal consent was obtained. A survey solicited information about each participant’s demographics, experiences with violence, and thoughts of harm to self or others. The survey was completed by 49 adolescents. The mean age was 16.78 (SD 2.34); 65% were female. Chi-Square, t-test, and Fisher’s exact were used to investigate the relationship between demographics, reported violence experiences and having self-harm thoughts. About half (45%) had experienced physical violence, 76% had experienced emotional violence, and 12% had experienced sexual violence. The most common perpetrators of physical and emotional violence were classmates, and the most common perpetrator of sexual violence was an ex-partner. Ten participants (20.4%) had thought about harming themselves. Self-harm thoughts were related to being female, employed, and to a higher number of experiences of physical and sexual violence. The results of this study suggest a high prevalence of both violence and self-harm thoughts in adolescents in the DR. Interventions that address physical and sexual violence against adolescents may be particularly important. Particular attention should also be paid to screening for self-harm thoughts in female-identifying adolescents. Further research is needed to better understand the relationship between violence and self-harm thoughts in adolescents in the DR.
**Opposed Reviewers:**	
**Additional Information:**	
**Question**	**Response**
**Financial Disclosure**	This project was self funded by Clínica de Familia La Romana and the authors.

Powered by Editorial Manager® and ProduXion Manager® from Aries Systems Corporation

Enter a financial disclosure statement that describes the sources of funding for the work included in this submission and the role the funder(s) played. This includes grants and any commercial funding of the work or authors.<br><br>This statement will be typeset if the manuscript is accepted for publication.<br><br>*Please review the submission guidelines and the instructions link below for detailed requirements and guidance.*	
**Competing Interests**<br><br>On behalf of all authors, disclose any competing interests that could be perceived to bias this work.<br><br>This statement will be typeset if the manuscript is accepted for publication.<br><br>*Please review the instructions link below and PLOS Global Public Health's competing interests policy to determine what information must be disclosed at submission.*	None of the author have any conflict of interest to declare.
**Data Availability**<br><br>Before publication, Authors are required to make fully available and without restriction all data underlying their findings. Please see our PLOS Data Policy page for detailed information on this policy.<br><br>A **Data Availability Statement**, detailing where the data can be accessed, is required at first submission. Insert your Data Availability Statement in the box	Dataset for this article will become available upon reasonable request.

below.<br><br>Please see the data reporting section of our submission guidelines for instructions on what you need to include in your Data Availability Statement.<br><br>This statement will be typeset if the manuscript is accepted for publication.<br><br>*PLOS allows rare exemptions to address legal and ethical concerns. If you have legal or ethical restrictions, please use the box below to detail these in full sentences for the Journal team to consider.*	

Powered by Editorial Manager® and ProduXion Manager® from Aries Systems Corporation

Clínica de Familia La Romana
Una organización de salud integral de la comunidad.

Dear Dr. Burgess and Dr. Chibanda,

We would like to submit a manuscript entitled "Thoughts of self-harm in adolescents: relationship with violence in the Dominican Republic" to be considered for publication as a research article in PLOS Global Public Health.

Our research focuses on factors associated with self-harm thoughts in adolescents between 14 and 20 years of age in the Dominican Republic; the factors explored which included age, female sex, employment status, and physical and sexual violence victimization. Previous studies have identified many of these factors as associated with self-harm thoughts and behaviors in adolescents, but the vast majority of these studies have taken place in high-income countries. There are very few studies examining factors associated with self-harm thoughts in Latin America and the Caribbean, and none, to our knowledge, in the Dominican Republic. Having evidence from Latin America and the Caribbean on the relationship between mental health and violence will help develop intervention that will be effective in this region. As such, we believe this article will be of interest to readers of PLOS Global Public Health.

For the reviewing process, we confirm to upload the dataset alongside the manuscripts and explored PLOSOne's editor board for potential reviewers, seeking individuals with experience in the topics of mental health, adolescents and self-harm thoughts. Thus, we propose individuals like Runsen Chen and Nafisa Halim, whom have worked on and produced research based on topics of self harm, suicidality and adolescent mental health. We have no opposed reviewers in mind. All authors have read and approved the final manuscript for submission.

We have had no previous contact with PLOS regarding this submission.

Thank you for considering our manuscript for publication in PLOS Global Public Health.

Sincerely,

Kelsey Badger
Columbia University Vagelos College of Physicians and Surgeons

Manuscript

Click here to access/download;Manuscript;PLoS Submission 5.8.23 Final.docx

Thoughts of self-harm in adolescents: relationship with violence in the Dominican Republic

Kelsey Badger[1], Pamela Baez Caraballo[2], Ahzyris Gibbs[2], Luz Messina[2], Mina Halpern[2], Silvia Amesty[3,4,5]

[1] Columbia University Vagelos College of Physicians and Surgeons, New York, NY, United States of America
[2] Clínica de Familia, La Romana, Dominican Republic
[3] Center for Family and Community Medicine, Columbia University Irving Medical Center, New York, NY, United States of America
[4] Columbia University Mailman School of Public Health, New York, NY, United States of America
[5] Department of Medical Humanities and Ethics, Columbia University Irving Medical Center, New York, NY, United States of America

*Corresponding author
E-mail: kkb2129@cumc.columbia.edu (KB)

Abstract

Violence against adolescents is a pressing health problem with long-term implications for future physical and mental well-being. One indication of poor mental well-being of adolescents is thoughts of self-harm, which are linked to suicidal ideation and completion. Although previous research has associated experiences of violence in childhood and adolescence with self-harm thoughts and behaviors, much of the research has been conducted only in high-income countries. This study aimed to examine the correlation between violence against adolescents and self-harm thoughts in the Dominican Republic (DR). Cross-sectional survey data was collected at a community-based clinic from participants aged 13-20. Participants were recruited through the clinic's adolescent program, and verbal consent was obtained. A survey solicited information about each participant's demographics, experiences with violence, and thoughts of harm to self or others. The survey was completed by 49 adolescents. The mean age was 16.78 (SD 2.34); 65% were female. Chi-Square, t-test, and Fisher's exact were used to investigate the relationship between demographics, reported violence experiences and having self-harm thoughts. About half (45%) had experienced physical violence, 76% had experienced emotional violence, and 12% had experienced sexual violence. The most common perpetrators of physical and emotional violence were classmates, and the most common perpetrator of sexual violence was an ex-partner. Ten participants (20.4%) had thought about harming themselves. Self-harm thoughts were related to being female, employed, and to a higher number of experiences of physical and sexual violence. The results of this study suggest a high prevalence of both violence and self-harm thoughts in adolescents in the DR. Interventions that address physical and sexual violence against adolescents may be particularly important. Particular attention should also be paid to screening for self-harm thoughts in female-identifying adolescents. Further research is needed to

better understand the relationship between violence and self-harm thoughts in adolescents in the DR.

Introduction

Violence against children is a major public health problem with an estimated one billion children having experienced sexual, physical, or emotional violence in 2016.[1] In Latin America and the Caribbean (LAC), homicide rates are amongst the highest in the world[2] and the leading cause of death among adolescents.[3] This age group is susceptible not only to physical violence, but also to sexual and dating violence, emotional violence, and bullying.[4] Up to one fifth of ever-partnered adolescent girls in LAC region have experienced interpersonal violence (IPV).[5] Violence experienced during adolescence has been linked to a number of negative mental health outcomes such as risky behavior, substance use, depression, post-traumatic stress, and self-directed violence.[6,7,8]

There is a wealth of data supporting the idea that exposure to violence in childhood and adolescents has a long-term negative impact on mental health. One of first studies to firmly establish the negative effects of exposure to violence in childhood and adolescence on long-term health outcomes was the adverse childhood experiences (ACE) study. This study showed that ACEs, which include childhood abuse and neglect and household dysfunction, are associated with poorer mental health and overall health as adults.[9] The majority of studies on the impacts of violence against adolescents' on mental health have been conducted in high-income countries and outside of LAC. In the Dominican Republic (DR), it has been estimated that around 80% of adolescents have experienced at least one ACE, with the most prevalent being physical abuse and witnessing domestic violence in their household.[10] This study also showed that in Dominican adolescents, having experienced more ACEs was associated with depression, anxiety, and dating

violence perpetration and victimization. Studies across the world support the link between experiences of violence in adolescence and poor mental health. One study that took place in Guatemala showed that in children and adolescents, exposure to violence increased rates of depression and anxiety.[11] Another study conducted in Vietnam showed that emotional violence in particular was associated with poor mental health.[12] Studies have also shown increased rates of suicidal thoughts, depression, and post-traumatic stress in adolescents that have experienced more than one type of violence.[13,14]

Self-harm thoughts have been linked with bullying and violence against adolescents,[15] and they are associated with increased risk of suicidal ideation and suicide completion.[16,17] Estimates of the prevalence of self-harm thoughts and behaviors vary widely. One large meta-analysis which included estimates from many different countries estimated the lifetime prevalence of self-harm behavior to be 17%.[18] One study from Chile found a 23% prevalence of self-harm among adolescents from low-income backgrounds.[19] However, there is limited data on the prevalence of self-harm thoughts and behaviors in the LAC region. Self-harm thoughts and suicidal behavior have been linked to violence experienced as children or adolescents.[20] One large study conducted in the United States found that emotional maltreatment and neglect in particular were associated with self-harm thoughts.[21] Another study found that suicidal ideation was associated with peer victimization, sexual assault, and maltreatment by a caregiver.[22] Peer victimization in particular has been linked to self-harm and suicidal ideation.[23,24] One study estimated the rate of bullying in the DR at 44%,[25] where bullying was defined as unwanted, aggressive behavior, which involves a real or perceived social power imbalance and is repeated over time.[26]

In sum, violence against adolescents is a public health problem that has major long-term impacts on both physical and mental health. There is very little information on the prevalence of violence against adolescents, self-harm thoughts, and the interaction between the two from low- and middle- income countries and from the LAC region. This study is, to our knowledge, the first to examine a relationship between violence against adolescents and self-harm thoughts in the DR.

Methods

This research study took place at a community-based clinic providing healthcare services to children, adolescents, and adults in La Romana, DR. The clinic has an adolescent program that focuses on sexual and reproductive health education, adolescent health services, and family planning. Services are delivered both at the clinic and through outreach at schools and other community centers where youth gather.

Data was collected from August to October 2022. A total of 49 participants aged 13-20 and residents of La Romana, DR were recruited by educators from the clinic's adolescent program. Verbal assent for adolescents younger than 18, and verbal consent for those 18 and older was obtained from each participant. For survey administration, questions were read by a member of the study team to small groups with no more than two to five participants at a time, as we have learned from previous studies done in this community that this is the best approach. Each adolescent filled their own paper questionnaire privately, in order to mitigate response bias. Participants recorded their answers on the paper survey, and the research assistant checked for completeness to avoid missing data, then answers were entered into REDCap, a secure web-based survey.[27,28] During data collection, authors involved in data collection could have identified individual participants. However, after surveys were entered into REDCap, individual

participants were not able to be identified. The questionnaire administration took place in a quiet, private space and took approximately 30 to 40 minutes to complete. This study received approval from Columbia University IRB and the Dominican Republic's National Health Bioethics Council (CONABIOS).

The questionnaire collected socio-demographic information, including age, sex, nationality, level of education, employment status, and composition of household. It also solicited information on experiences with physical, sexual, and emotional/psychological violence, on participants' attitudes about gender and violence, and on help-seeking behaviors and thoughts of harm toward self and others.

Each type of violence - physical, sexual, and emotional/psychological - was defined for participants. They were first asked if they had ever experienced that type of violence. All participants were asked about experiences regarding specific violent acts: for example, for physical violence, participants were asked to indicate if they had experienced any of the following: been slapped, hit, or kicked; been a victim of attempted kidnapping; been stabbed; been tied up or strangled; or been threatened with a firearm. If participants reported experiencing violent acts, they were asked who perpetrated these acts of violence. Participants were asked about perpetrating violent acts against others, the types of violence committed, and who they had committed the act against.

The questionnaire also included questions about help seeking behaviors. Participants were asked if they currently felt vulnerable to violence, if they had anyone to talk to about experiences with violence, and if they knew where to go if they were a victim of violence. At the end, they were asked if they had ever thought about hurting themselves or someone else, then asked to indicate who they had thought about hurting and if they had thought about seeking help

for those thoughts. Psychological support services were available at the clinic for those who were interested.

Analysis

We explored types of violence experienced or perpetrated by demographic characteristics. We examined whether participants experienced any type of violence perpetrated by others or committed acts of violence. We looked at self-harm thoughts, and types of violence reported by adolescents, those reporting self-harm thoughts and those who did not.

We began analysis with descriptive statistics of the population, to view distribution by demographic variables, and establish prevalence of suffering by each type of violence. Both the rates of violence by self-report and by rate of having experienced a listed act of violence were reported. For bivariable analysis, tests of association were used including Chi-squared test (when appropriate), Fisher's exact test (when appropriate), and two-sample t-test (when comparing two means). A value of $p < 0.05$ was set for statistical significance. The associations were test between having self-harm thoughts, participant demographics and experiencing violence.

Results

Demographics

A total of 49 adolescents completed the survey (Table 1a). The mean age was 16.78 (SD 2.34). Participants were mainly Dominican, female, and from urban areas. More than half had not yet to complete high school (55.1%), and one fifth of participants had dropped out of school at some point (18.4%). About 26.5% of participants lived with both parents and sibling(s), and 40.8% lived with one parent. A quarter of the participants were currently employed.

**Table 1a:** Sample characteristics (n=49)	
Variable	Frequency (%)
Where participants live	
Rural area	2 (4.1)
Urban area	47 (95.9)
Mean age	16.78
Sex	
Male	16 (32.7)
Female	32 (65.3)
Did not know	1 (2.0)
Country of origin	
Dominican	49 (100)
Level of education	
Not completed high school	27 (55.1)
Completed high school	22 (44.9)
Ever dropped out of school	
Yes	9 (18.4)
No	39 (79.6)
No response	1 (2.0)
Lives with	
Both parents and siblings	13 (26.5)
One parent	20 (40.8)
Other close family (grandparents, siblings)	13 (26.5)
Other extended family (uncle, step father)	6 (12.2)
Friends	1 (2.0)
Other	5 (10.2)
Currently employed	
Yes	12 (24.5)
No	37 (75.5)

Thoughts of harm

Eighteen participants, or 36.7%, had thoughts of causing harm to themselves or others (Table 1b), and 20.4% had thought about harming themselves. Almost a quarter of the total sample had thought about seeking help for these thoughts about harming themselves or others.

Table 1b: Thoughts of harm (n=49)	
Variable	Frequency (%)
Ever thought about causing harm to self or others	
Yes	18 (36.7)
No	27 (55.1)
No response	4 (8.2)
Thought about harming	
Current partner	1 (2.0)
Family member other than father	4 (8.2)
Classmate	3 (6.1)
Self	10 (20.4)
No response	2 (4.1)
Other	1 (2.0)
Ever thought about seeking help for thoughts of harm to self or others	
Yes	12 (24.5)
No	5 (10.2)
No response	1 (2.0)

Violence victimization

Almost one third of the participants (30.6%) reported having been a victim of physical violence (Table 2a). When asked if they had experienced any of the acts of physical violence listed in the questionnaire, close to half of participants reported having experienced physical violence (44.9%). The most common perpetrator of physical violence was a classmate, with 12.2% of participants having experienced physical violence committed by a classmate. Almost one in 10 had experienced physical violence at the hands of a stranger (8.2%). When emotional violence was defined, 44.9% of participants reported experiencing emotional violence. However, when asked if they had experienced specific acts of emotional violence ("has anyone ever made you feel embarrassed or humiliated?", "Has anyone ever insulted you?"), three quarters of the sample indicated that they had (75.5%), and 24.5% reported experiencing emotional violence perpetrated by a classmate, an ex-partner (20.4%), or a family member that was not their father (14.3%). About one in 10 participants reported experiencing sexual violence (12.2%). When asked about specific acts of sexual violence, seven of the 49 participants (14.3%) reported having experienced at least one act of sexual violence. Of the seven participants that experienced sexual

violence, three of them (42.9%) experienced violence perpetrated by a partner or ex-partner and three (42.9%) of them chose not to indicate who committed the act against them.

Table 2a: Violence victimization (n=49)

Variable	Frequency (%)
Number of types of violence experienced	
0	10 (20.4)
1	18 (36.7)
2	15 (30.6)
3	6 (12.3)
Mean number of types of violence experienced	1.35
Ever experienced physical violence (self-report)	
Yes	15 (30.6)
No	32 (65.4)
Did not know	1 (2.0)
No response	1 (2.0)
Ever experienced act of physical violence	
Yes	22 (44.9)
No	27 (55.1)
Experienced physical violence perpetrated by	
Ex-partner	3 (6.1)
Father	3 (6.1)
Other family member	3 (6.1)
Classmate	6 (12.2)
No response	1 (2.0)
Stranger	4 (8.2)
Other	3 (6.1)
Ever experienced emotional violence (self-report)	
Yes	22 (44.9)
No	25 (51.1)
Did not know	1 (2.0)
No response	1 (2.0)
Ever experienced an act of emotional violence	
Yes	37 (75.5)
No	12 (24.5)
Perpetrator of emotional violence	
Current partner	3 (6.1)
Ex-partner	10 (20.4)
Father	4 (8.2)
Other family member	7 (14.3)
Classmate	12 (24.5)
Teacher	1 (2.0)
No response	7 (14.3)
Stranger	2 (4.1)
Other	2 (4.1)
Ever experienced sexual violence (self-report)	
Yes	6 (12.2)
No	41 (83.7)
No response	2 (4.1)
Experienced an act of sexual violence	
Yes	7 (14.3)
No	42 (85.7)
Perpetrator of sexual violence	
Current partner	1 (2.0)
Ex-partner	2 (4.1)
No response	3 (6.1)
Other	1 (2.0)

Violence perpetration

More than one third of participants admitted to having committed an act of physical violence (34.7%; Table 2b). Almost one fifth of participants indicated that they had committed

an act of physical violence against a classmate (18.4%). More than half of participants reported having committed an act of emotional violence (53.1%) and close to one quarter of participants had committed an act of emotional violence against a classmate (22.5%). Two of the participants (4.1%) reported having committed an act of sexual violence.

Table 2b: Violence perpetration (n=49)

Variable	Frequency (%)
Committed an act of physical violence	
Yes	17 (34.7)
No	32 (65.3)
Committed an act of physical violence against	
Ex-partner	2 (4.1)
Other family member	2(4.1)
Classmate	9 (18.4)
No response	4 (8.2)
Stranger	1 (2.0)
Other	2 (4.1)
Committed an act of emotional violence	
Yes	26 (53.1)
No	23 (46.9)
Committed an act of emotional violence against	
Current partner	1 (2.0)
Ex-partner	3 (6.1)
Father	1 (2.0)
Other family member	4 (8.2)
Authority figure (police, etc.)	1 (2.0)
Classmate	11 (22.5)
Teacher	1 (2.0)
No response	4 (8.2)
Stranger	2 (4.1)
Other	3 (6.1)
Committed an act of sexual violence	
Yes	2 (4.1)
No	47 (95.9)
Committed an act of sexual violence against	
Classmate	1 (2.0)
No response	1 (2.0)

Bivariate analysis

All of the participants reporting self-harm thoughts were female (p=0.025; Table 3), mean age 18 (p=0.053). None of the participants reporting self-harm thoughts lived with both parents, compared to one third (33.3%) of those reporting no self-harm thoughts (p=0.033). Half of those reporting self-harm thoughts were currently working compared to only 7.7% of those who reported no self-harm thoughts (p=0.050).

The mean number of types of violence suffered by those reporting self-harm thoughts was significantly higher than those reporting no self-harm thoughts, 2.20 vs. 1.13 types of violence (p=0.010). The vast majority (80%) of participants reporting self-harm thoughts had experienced physical violence compared to 35.9% of those reporting no self-harm thoughts (p=0.029). Half of those reporting self-harm thoughts had experienced sexual violence (p=0.0023), compared to 5.1% of those reporting no self-harm thoughts.

**Table 3**: Bivariate Analysis			
Variable	Frequency without self-harm thoughts (%) n=39	Frequency with self-harm thoughts (%) n=10	p-value
Sex			
Male	16 (41.0)	0 (0.0)	***0.025***
Female	22 (56.4)	10 (100.0)	
Did not know	1 (2.6)	0 (0.0)	
Mean age	16.46	18.00	0.053
Level of coursework completed			
Not completed high school	22 (56.4)	5 (50.0)	0.72
Completed high school	17 (43.6)	5 (50.0)	
Ever dropped out of school			
Yes	7 (7.7)	2 (20.0)	1
No	31 (79.5)	8 (80.0)	
No response	1 (2.6)	0 (0.0)	
Lives with both parents	13 (33.3)	0 (0.0)	***0.033***
Does not live with both parents	26 (66.7)	10 (100.0)	
Currently working			
Yes	7 (7.7)	5 (50.0)	***0.050***
No	32 (82.1)	5 (50.0)	
Ever experienced any violence			
Yes	30 (76.9)	9 (90.0)	0.66
No	9 (23.1)	1 (10.0)	
Mean number of types of violence experienced	1.13	2.20	***0.010***
Ever experienced physical violence			
Yes	14 (35.9)	8 (80.0)	***0.029***
No	25 (64.1)	2 (20.0)	
Ever perpetrated physical violence			
Yes	14 (35.9)	3 (30.0)	1
No	25 (64.1)	7 (70.0)	
Ever experienced emotional violence			
Yes	28 (71.8)	9 (90.0)	0.41
No	11 (28.2)	1 (10.0)	
Ever perpetrated emotional violence			
Yes	21 (53.9)	5 (50.0)	1
No	18 (46.2)	5 (50.0)	
Ever experienced sexual violence			
Yes	2 (5.1)	5 (50.0)	***0.0023***
No	37 (94.9)	5 (50.0)	
Ever perpetrated sexual violence			
Yes	1 (2.6)	1 (10.0)	0.37
No	38 (97.4)	9 (90.0)	

Subgroup analysis for female-identifying participants

On our bivariate analysis including only female-identifying participants, we found several factors correlated with having thoughts of self-harm. The mean age of those reporting

self-harm thoughts was 18, compared to 16.3 for those reporting no self-harm thoughts (p=0.053; Table 4). None of the female-identifying participants who reported self-harm thoughts lived with both parents, compared to 36.3% of those who reported no self-harm thoughts (p=0.035). Half of those reporting self-harm thoughts were working at the time the survey was administered, compared to 9.1% of those reporting no self-harm thoughts (p=0.019). Participants reporting self-harm thoughts had experienced an average number of 2.2 different types of violence compared to those reporting no self-harm thoughts (1.1 different types of violence) (p=0.0075). Eighty percent of participants reporting self-harm thoughts had experienced physical violence compared to 22.7% of those reporting no self-harm thoughts (p=0.0051). Half of those reporting self-harm thoughts had experienced sexual violence, compared to 9.1% of those reporting no self-harm thoughts (p=0.0023).

Table 4: Bivariate Analysis for Female-Identifying Participants Only

Variable	Frequency without self-harm thoughts (%) n=22	Frequency with self-harm thoughts (%) n=10	p-value
Mean age	16.31	18.00	***0.053***
Level of coursework completed			
Not completed high school	14 (63.7)	5 (50.0)	0.70
Completed high school	8 (36.3)	5 (50.0)	
Ever dropped out of school			
Yes	4 (18.2)	2 (20.0)	1
No	18 (81.8))	8 (80.0)	
Lives with both parents	8 (36.3)	0 (0.0)	***0.035***
Does not live with both parents	14 (63.7)	10 (100.0)	
Currently working			
Yes	2 (9.1)	5 (50.0)	***0.019***
No	20 (90.9)	5 (50.0)	
Ever experienced any violence			
Yes	16 (72.7)	9 (90.0)	0.39
No	6 (27.3)	1 (10.0)	
Mean number of types of violence experienced	1.05	2.20	***0.0075***
Ever experienced physical violence			
Yes	5 (22.7)	8 (80.0)	***0.0051***
No	17 (77.3)	2 (20.0)	
Ever perpetrated physical violence			
Yes	6 (27.3)	3 (30.0)	1
No	16 (72.7)	7 (70.0)	
Ever experienced emotional violence			
Yes	16 (72.7)	9 (90.0)	0.41
No	6 (27.3)	1 (10.0)	
Ever perpetrated emotional violence			
Yes	13 (59.1)	5 (50.0)	1
No	9 (40.9)	5 (50.0)	
Ever experienced sexual violence			
Yes	2 (9.1)	5 (50.0)	***0.0023***
No	20 (90.9)	5 (50.0)	
Ever perpetrated sexual violence			
Yes	0 (0.0)	1 (10.0)	0.37
No	22 (100.0)	9 (90.0)	

Discussion

This study aimed to examine the relationship between violence against Dominican adolescents and self-harm thoughts. Our results are consistent with previous studies which have shown higher rates of self-harm thoughts and behaviors in females.[18,29] This study is one of the first, to our knowledge, which provides data on prevalence of thoughts of self-harm in

Dominican adolescents. With a rate of nearly one third of female-identifying adolescents indicating having thought of self-harm, this indicates a great need for mental health support for this population. Agencies that provide services to this population should screen for mental health problems, especially self-harm thoughts which could indicate risk of suicide.

None of the males in our study reported having thoughts of self-harm, which could be due to under-reporting. Studies have shown that those who identify as male tend to under-report mental health issues.[30] There is a body of evidence showing that males seek help for mental health problems at lower rates than females,[31,32] and that they have lower mental health literacy than females.[33,34] Some studies have also suggested that men perceive more mental health stigma and that this effect is particularly prominent in younger men.[35] Future work to reduce the stigma associated with mental illness and increasing awareness could result in higher reporting among male adolescents.

It has been reported that sexual assault against adolescent females significantly contributes to the higher rate of self-harm thoughts and behaviors in adolescent females.[36] Our results are supported by evidence that sexual violence victimization is associated with high rates of self-harm thoughts and behaviors.[37,38] This shows the profound impact that experiences with sexual violence can have on the mental health of adolescent girls in the DR. Our study also found that the most common perpetrators of sexual violence were current or former romantic partners, indicating that adolescent dating violence (ADV) may have a significant impact on the mental health of adolescent females. ADV is defined as a type of intimate partner violence that can include sexual, physical, or psychological abuse that occurs between two adolescents in a close relationship.[39] Interventions that address ADV could decrease the rates of sexual violence and mental health conditions in Dominican adolescents. Studies have shown that interventions

aiming to reduce adolescent dating violence are effective in reducing physical violence and possibly sexual violence among adolescents.[40] Interventions to reduce sexual violence are still a developing area of study, and to our knowledge, there are no studies evaluating these interventions in the DR and very few in Latin America. Our study may underestimate the rate of sexual violence for adolescents in the DR because of the stigma associated with sexual violence victimization.

We found that for physical violence, which was also associated with self-harm thoughts, the most common perpetrator was a classmate. The high prevalence of violence amongst peers adds to a growing body of evidence that suggests the need for increased intervention in schools and other places where adolescents gather aimed at decreasing physical and emotional violence in particular. In addition to physical violence perpetrated by peers, several participants in our study also reported violence perpetrated by family members. Familial abuse has been linked to higher rate of self-harm thoughts in adolescents by several studies.[21,22] When it comes to physical violence, both peer victimization and familial violence are important factors in self-harm thoughts.

Previous research has shown that adolescents who live in blended households with step-parents have higher rates of self-harm behavior.[41] Non-suicidal self-injury has also been linked to family events such as divorce or death of one parent.[42] This association should be explored further in future studies in order to better understand this potential risk factor.

We found that participants with self-harm thoughts in our study were, on average, older than those without self-harm thoughts. Generally, adolescence is when self-harm behaviors peak, with a decline in prevalence in early adulthood.[43,44] It is also unclear whether age and employment status were independent risk factors in our study or if because participants with self-

harm thoughts were older, they were more likely to be working at the time of the survey. It could also be that the adolescents in our study that were working were from lower-income households where they were required to work. Previous studies have established that adolescents from low-income backgrounds have higher rates of self-harm.[45,46]

Our study found a difference in responses to questions about violence when questions were asked differently. Participants in this study were asked about violence in two ways: asking if adolescents had experienced a type of violence after providing a definition of the violence and after providing a list of acts of violence. More participants answered affirmatively after being provided a list of violent acts. It may be necessary to limit abstraction when answering questions and ask about concrete experiences, particularly when working with adolescents.[47] The difference was especially striking for emotional violence, where almost twice the number of participants responded that they had experienced emotional violence after they were provided with a list of acts. This highlights the need to carefully define types of violence in a concrete way in surveys in order to avoid underestimating rates of violence in this population.

Limitations

The most significant limitation in this study is its small sample size. The small sample size limited our options for what types of analysis could be conducted. In addition, since this study was done with a cross-sectional questionnaire, it was susceptible to response and recall bias. Violence tends to be under-reported because experiencing violence is traumatic and stigmatizing, especially for adolescents. The questionnaire may have also been subject to desirability bias, since they were conducted in-person and participants may have been tempted to answer questions in a way that they felt would please the interviewer. To prevent this, however,

we encouraged the adolescents to fill out the surveys themselves, and each type of violence was defined by the research assistant prior to survey completion, for the sake of accuracy.

Although we know that bullying is highly prevalent in the DR, it is impossible to draw conclusions about bullying from this study, since bullying is defined as a repeated behavior. Our questionnaire did not have a component that addressed this aspect of violence. In addition, types of relational violence such as spreading rumors or excluding someone from a group, were not measured by the questionnaire. This type of violence is more likely to affect girls.[48] Future studies could explore bullying in this population by asking about repeated behavior and other specific types of violence.

Another limitation is that our questionnaire asked only if participants had self-harm thoughts, not if these thoughts were suicidal or not suicidal. Our questionnaire also did not ask if participants had acted on any of these thoughts in the form of non-suicidal self-harm or suicide attempts. Future studies could explore whether or not participants have engaged in self-harm behavior or had depressive symptomatology in order to better understand risk for suicide and prevalence of non-suicidal self-harm.

Conclusions

More research is needed to understand the patterns of violence against adolescents and self-harm thoughts in adolescents in the DR. Our findings add to a larger body of evidence suggesting a relationship between violence and self-harm thoughts and poor mental health in general. This study is the first, to our knowledge, to attempt to define this relationship for Dominican adolescents, and there is still a need for more research in this field. Sexual violence against adolescents, especially female adolescents, is of particular concern, and more work can be done to prevent and screen for sexual violence in order to identify adolescents at risk for

mental health problems. Those that care for youth should consider youth experiences with violence and mental health when evaluating them. This study highlights the need for education about adolescent dating violence, which could help be effective in decreasing sexual violence against adolescents and therefore, promoting mental wellness. Further research is needed to fully characterize self-harm thoughts, behaviors, suicidality, and depressive symptomatology in adolescents from the DR. The high prevalence of peer-to-peer violence in our study highlights a need to address peer violence and bullying and further examine its effects on the mental health of Dominican adolescents.

Acknowledgements

We would like to thank the staff at Clínica de Familia La Romana for contributing significant time to this project. This study would not have been possible without their support. We also thank Columbia University for project funding and guidance. Above all, we thank the participants in this study for their bravery in sharing their experiences with us.

Funding

This project was funded by the Columbia University Vagelos College of Physicians and Surgeons Scholarly Projects program and the Friedman award.

We have no known conflicts of interest to disclose.

References

1. Hillis S, Mercy J, Amobi A, Kress H. Global Prevalence of Past-year Violence Against Children: A Systematic Review and Minimum Estimates. Pediatrics. 2016;137(3):e20154079. doi:10.1542/peds.2015-4079.
2. Muggah R, Aguirre K. Citizen Security in Latin America: Facts and figures, Igarapé Institute, 2018. Available from: https://igarape.org.br/en/citizen-security-in-latin-america-facts-and-figures/.

3. United Nations Children's Fund. A familiar face: Violence in the lives of children and adolescents [Internet]. New York: UNICEF; 2017 [cited 2023 Apr 21]. Available from: https://data.unicef.org/topic/child-protection/violence/.
4. Violence Against Children and Youth Surveys. CDC. https://www.cdc.gov/violenceprevention/childabuseandneglect/vacs/index.html.
5. Devries, K., Merrill, K. G., Knight, L., Bott, S., Guedes, A., Butron-Riveros, B., Hege, C., Petzold, M., Peterman, A., Cappa, C., Maxwell, L., Williams, A., Kishor, S., & Abrahams, N. Violence against children in Latin America and the Caribbean: What do available data reveal about prevalence and perpetrators?. Revista panamericana de salud publica = Pan American journal of public health. 2019; 43, e66. https://doi.org/10.26633/RPSP.2019.66.
6. Holmes K, Sher L. Dating violence and suicidal behavior in adolescents. Int J Adolesc Med Health. 2013;25(3):257-261. doi:10.1515/ijamh-2013-0059
7. Duke NN, Pettingell SL, McMorris BJ, Borowsky IW. Adolescent violence perpetration: associations with multiple types of adverse childhood experiences. Pediatrics. 2010 Apr;125(4):e778-86. doi: 10.1542/peds.2009-0597. Epub 2010 Mar 15. PMID: 20231180.
8. Exner-Cortens D, Eckenrode J, Rothman E. Longitudinal associations between teen dating violence victimization and adverse health outcomes. Pediatrics. 2013 Jan;131(1):71-8. doi: 10.1542/peds.2012-1029. Epub 2012 Dec 10. PMID: 23230075; PMCID: PMC3529947.
9. Felitti VJ, Anda RF, Nordenberg D, Williamson DF, Spitz AM, Edwards V, Koss MP, Marks JS. Relationship of childhood abuse and household dysfunction to many of the leading causes of death in adults. The Adverse Childhood Experiences (ACE) Study. Am J Prev Med. 1998 May;14(4):245-58. doi: 10.1016/s0749-3797(98)00017-8. PMID: 9635069.
10. Luft HS, Mersky JP, Choi C, Canario Guzmán JA, Quezada Ortiz MV, Sehi GT, Temple JR. Prevalence of adverse childhood experiences (ACEs) and association with dating violence and symptoms of mental illness among adolescents in the Dominican Republic. Child Abuse Negl. 2022 Jul;129:105668. doi: 10.1016/j.chiabu.2022.105668. Epub 2022 May 23. PMID: 35617753.
11. Company-Córdoba R, Gómez-Baya D, López-Gaviño F, Ibáñez-Alfonso JA. Mental Health, Quality of Life and Violence Exposure in Low-Socioeconomic Status Children and Adolescents of Guatemala. Int J

Environ Res Public Health. 2020 Oct 19;17(20):7620. doi: 10.3390/ijerph17207620. PMID: 33086701; PMCID: PMC7589969.

12. Nguyen HT, Dunne MP, Le AV. Multiple types of child maltreatment and adolescent mental health in Viet Nam. Bull World Health Organ. 2010 Jan;88(1):22-30. doi: 10.2471/BLT.08.060061. Epub 2009 Sep 2. Erratum in: Bull World Health Organ. 2010 Mar;88(3):236. PMID: 20428350; PMCID: PMC2802435.
13. Kamndaya M, Pisa PT, Chersich MF, Decker MR, Olumide A, Acharya R, Cheng Y, Brahmbhatt H, Delany-Moretlwe S. Intersections between polyvictimisation and mental health among adolescents in five urban disadvantaged settings: the role of gender. BMC Public Health. 2017 Jul 4;17(Suppl 3):525. doi: 10.1186/s12889-017-4348-y. PMID: 28832286; PMCID: PMC5498854.
14. Chan KL. Victimization and poly-victimization among school-aged Chinese adolescents: prevalence and associations with health. Prev Med. 2013 Mar;56(3-4):207-10. doi: 10.1016/j.ypmed.2012.12.018. Epub 2013 Jan 4. PMID: 23295171
15. Stallard P, Spears M, Montgomery AA, Phillips R, Sayal K. Self-harm in young adolescents (12-16 years): onset and short-term continuation in a community sample. BMC Psychiatry. 2013 Dec 2;13:328. doi: 10.1186/1471-244X-13-328. PMID: 24294921; PMCID: PMC4219511.
16. Baldwin JR, Arseneault L, Caspi A, Moffitt TE, Fisher HL, Odgers CL, Ambler A, Houts RM, Matthews T, Ougrin D, Richmond-Rakerd LS, Takizawa R, Danese A. Adolescent Victimization and Self-Injurious Thoughts and Behaviors: A Genetically Sensitive Cohort Study. J Am Acad Child Adolesc Psychiatry. 2019 May;58(5):506-513. doi: 10.1016/j.jaac.2018.07.903. Epub 2018 Dec 12. PMID: 30768402; PMCID: PMC6494951.
17. Castellví P, Lucas-Romero E, Miranda-Mendizábal A, Parés-Badell O, Almenara J, Alonso I, Blasco MJ, Cebrià A, Gabilondo A, Gili M, Lagares C, Piqueras JA, Roca M, Rodríguez-Marín J, Rodríguez-Jimenez T, Soto-Sanz V, Alonso J. Longitudinal association between self-injurious thoughts and behaviors and suicidal behavior in adolescents and young adults: A systematic review with meta-analysis. J Affect Disord. 2017 Jun;215:37-48. doi: 10.1016/j.jad.2017.03.035. Epub 2017 Mar 12. PMID: 28315579.
18. Gillies D, Christou MA, Dixon AC, Featherston OJ, Rapti I, Garcia-Anguita A, Villasis-Keever M, Reebye P, Christou E, Al Kabir N, Christou PA. Prevalence and Characteristics of Self-Harm in Adolescents:

Meta-Analyses of Community-Based Studies 1990-2015. J Am Acad Child Adolesc Psychiatry. 2018 Oct;57(10):733-741. doi: 10.1016/j.jaac.2018.06.018. Epub 2018 Aug 21. PMID: 30274648.

19. Spears M, Montgomery AA, Gunnell D, Araya R. Factors associated with the development of self-harm amongst a socio-economically deprived cohort of adolescents in Santiago, Chile. Soc Psychiatry Psychiatr Epidemiol. 2014 Apr;49(4):629-37. doi: 10.1007/s00127-013-0767-y. Epub 2013 Oct 6. PMID: 24097260; PMCID: PMC3969808.
20. Angelakis I, Austin JL, Gooding P. Association of Childhood Maltreatment With Suicide Behaviors Among Young People: A Systematic Review and Meta-analysis. JAMA Netw Open. 2020 Aug 3;3(8):e2012563. doi: 10.1001/jamanetworkopen.2020.12563. PMID: 32756929; PMCID: PMC7407092.
21. Turner HA, Colburn D. Independent and Cumulative Effects of Recent Maltreatment on Suicidal Ideation and Thoughts of Self-harm in a National Sample of Youth. J Adolesc Health. 2022 Feb;70(2):329-335. doi: 10.1016/j.jadohealth.2021.09.022. Epub 2021 Oct 19. PMID: 34674929
22. Turner HA, Finkelhor D, Shattuck A, Hamby S. Recent victimization exposure and suicidal ideation in adolescents. Arch Pediatr Adolesc Med. 2012 Dec;166(12):1149-54. doi: 10.1001/archpediatrics.2012.1549. PMID: 23090641.
23. Chen QQ, Chen MT, Zhu YH, Chan KL, Ip P. Health correlates, addictive behaviors, and peer victimization among adolescents in China. World J Pediatr. 2018 Oct;14(5):454-460. doi: 10.1007/s12519-018-0158-2. Epub 2018 Jun 28. PMID: 29956126.
24. Arseneault L. Annual Research Review: The persistent and pervasive impact of being bullied in childhood and adolescence: implications for policy and practice. J Child Psychol Psychiatry. 2018 Apr;59(4):405-421. doi: 10.1111/jcpp.12841. Epub 2017 Nov 14. PMID: 29134659; PMCID: PMC6542665.
25. Hosozawa M, Bann D, Fink E, Elsden E, Baba S, Iso H, Patalay P. Bullying victimisation in adolescence: prevalence and inequalities by gender, socioeconomic status and academic performance across 71 countries. EClinicalMedicine. 2021 Oct 11;41:101142. doi: 10.1016/j.eclinm.2021.101142. PMID: 34693231; PMCID: PMC8517283.
26. Waseem M, Nickerson AB. Bullying. 2022 Aug 7. In: StatPearls [Internet]. Treasure Island (FL): StatPearls Publishing; 2022 Jan–. PMID: 28722959.

27. Harris PA, Taylor R, Thielke R, Payne J, Gonzalez N, Conde JG, Research electronic data capture (REDCap) – A metadata-driven methodology and workflow process for providing translational research informatics support, J Biomed Inform. 2009 Apr;42(2):377-81.

28. Harris PA, Taylor R, Minor BL, Elliott V, Fernandez M, O'Neal L, McLeod L, Delacqua G, Delacqua F, Kirby J, Duda SN. REDCap Consortium. The REDCap consortium: Building an international community of software partners, J Biomed Inform. 2019 May 9. doi: 10.1016/j.jbi.2019.103208.

29. Fox KR, Millner AJ, Mukerji CE, Nock MK. Examining the role of sex in self-injurious thoughts and behaviors. Clin Psychol Rev. 2018 Dec;66:3-11. doi: 10.1016/j.cpr.2017.09.009. Epub 2017 Sep 28. PMID: 28993102.

30. Shi P, Yang A, Zhao Q, Chen Z, Ren X, Dai Q. A Hypothesis of Gender Differences in Self-Reporting Symptom of Depression: Implications to Solve Under-Diagnosis and Under-Treatment of Depression in Males. Front Psychiatry. 2021 Oct 25;12:589687. doi: 10.3389/fpsyt.2021.589687. PMID: 34759845; PMCID: PMC8572815.).

31. Merikangas KR, He JP, Burstein M, Swendsen J, Avenevoli S, Case B, Georgiades K, Heaton L, Swanson S, Olfson M. Service utilization for lifetime mental disorders in U.S. adolescents: results of the National Comorbidity Survey-Adolescent Supplement (NCS-A). J Am Acad Child Adolesc Psychiatry. 2011 Jan;50(1):32-45. doi: 10.1016/j.jaac.2010.10.006. Epub 2010 Dec 3. PMID: 21156268; PMCID: PMC4408275

32. Slade T, Johnston A, Oakley Browne MA, Andrews G, Whiteford H. 2007 National Survey of Mental Health and Wellbeing: methods and key findings. Aust N Z J Psychiatry. 2009 Jul;43(7):594-605. doi: 10.1080/00048670902970882. PMID: 19530016

33. Clark LH, Hudson JL, Rapee RM, Grasby KL. Investigating the impact of masculinity on the relationship between anxiety specific mental health literacy and mental health help-seeking in adolescent males. J Anxiety Disord. 2020 Dec;76:102292. doi: 10.1016/j.janxdis.2020.102292. Epub 2020 Sep 3. PMID: 33010663.

34. Bruno M, McCarthy J, Kramer C. Mental health literacy and depression among older adolescent males. Journal of Asia Pacific Counseling. 2015 August. doi: 10.18401/2015.5.2.1.

35. Mackenzie, C. S., Visperas, A., Ogrodniczuk, J. S., Oliffe, J. L., & Nurmi, M. A. (2019). Age and sex differences in self-stigma and public stigma concerning depression and suicide in men. Stigma and Health, 4(2), 233–241. https://doi.org/10.1037/sah0000138.
36. Bentivegna F, Patalay P. The impact of sexual violence in mid-adolescence on mental health: a UK population-based longitudinal study. Lancet Psychiatry. 2022 Nov;9(11):874-883. doi: 10.1016/S2215-0366(22)00271-1. Epub 2022 Oct 4. PMID: 36206779; PMCID: PMC9630148.
37. Quarshie EN. Self-Harm Among School-Going Adolescent Survivors of Sexual Violence Victimisation: A Cross-Sectional Study. Front Sociol. 2021 May 20;6:605865. doi: 10.3389/fsoc.2021.605865. PMID: 34095285; PMCID: PMC8174787.
38. Chang EC, Lee J, Wright KM, Najarian AS, Yu T, Chang OD, Hirsch JK. Examining Sexual Assault Victimization and Loneliness as Risk Factors Associated With Nonlethal Self-Harm Behaviors in Female College Students: Is It Important to Control for Concomitant Suicidal Behaviors (and Vice Versa)? J Interpers Violence. 2019 Nov;34(21-22):4443-4458. doi: 10.1177/0886260516675920. Epub 2016 Oct 26. PMID: 27784832.
39. US Centers for Disease Control and Prevention. Preventing teen dating violence [Internet]. 2020. Available from: https://www.cdc.gov/violenceprevention/pdf/ipv/TDV-factsheet_2020.pdf.
40. Piolanti A, Foran HM. Efficacy of Interventions to Prevent Physical and Sexual Dating Violence Among Adolescents: A Systematic Review and Meta-analysis. JAMA Pediatr. 2022 Feb 1;176(2):142-149. doi: 10.1001/jamapediatrics.2021.4829. PMID: 34842911; PMCID: PMC8630665.
41. de Kloet L, Starling J, Hainsworth C, Berntsen E, Chapman L, Hancock K. Risk factors for self-harm in children and adolescents admitted to a mental health inpatient unit. Aust N Z J Psychiatry. 2011 Sep;45(9):749-55. doi: 10.3109/00048674.2011.595682. Epub 2011 Aug 10. PMID: 21827346.
42. Martin J, Bureau JF, Yurkowski K, Fournier TR, Lafontaine MF, Cloutier P. Family-based risk factors for non-suicidal self-injury: Considering influences of maltreatment, adverse family-life experiences, and parent-child relational risk. J Adolesc. 2016 Jun;49:170-80. doi: 10.1016/j.adolescence.2016.03.015. Epub 2016 Apr 14. PMID: 27086083.
43. Gandhi A, Luyckx K, Baetens I, Kiekens G, Sleuwaegen E, Berens A, Maitra S, Claes L. Age of onset of non-suicidal self-injury in Dutch-speaking adolescents and emerging adults: An event history analysis of

pooled data. Compr Psychiatry. 2018 Jan;80:170-178. doi: 10.1016/j.comppsych.2017.10.007. Epub 2017 Oct 20. PMID: 29121554.

44. Plener PL, Schumacher TS, Munz LM, Groschwitz RC. The longitudinal course of non-suicidal self-injury and deliberate self-harm: a systematic review of the literature. Borderline Personal Disord Emot Dysregul. 2015 Jan 30;2:2. doi: 10.1186/s40479-014-0024-3. PMID: 26401305; PMCID: PMC4579518.
45. Park H, In S, Hur JW. Association of socioeconomic status with nonsuicidal self-injury and suicidal ideation in young Korean adults. Int J Soc Psychiatry. 2022 Aug;68(5):1127-1134. doi: 10.1177/00207640221104691. Epub 2022 Jun 14. PMID: 35699657.
46. Wang Q, Xiong Y, Liu X. Family Unity or Money? The Roles of Parent-Child Cohesion and Socioeconomic Status in the Relationship Between Stressful Life Events and Nonsuicidal Self-Injury Among Chinese Left-Behind Children. Suicide Life Threat Behav. 2020 Oct;50(5):963-974. doi: 10.1111/sltb.12650. Epub 2020 Jun 21. PMID: 32564411.
47. Omrani A, Scurr J, Smith J, Brown N. Survey Development for Adolescents Aged 11-16 Years: A Developmental, Science Based Guide. Adolescent Research Review. December 2019. Doi: 10.1007/s40894-018-0089-0.
48. Wang J, Iannotti RJ, Nansel TR. School bullying among adolescents in the United States: physical, verbal, relational, and cyber. J Adolesc Health. 2009 Oct;45(4):368-75. doi: 10.1016/j.jadohealth.2009.03.021. Epub 2009 Jun 11. PMID: 19766941; PMCID: PMC2751860.)

Author contributions

Kelsey Badger: conceptualization, formal analysis, funding acquisition, investigation, methodology, writing- original draft preparation, writing – review & editing

Pamela Baez Caraballo: conceptualization, data curation, investigation, methodology, project administration, resources, supervision, writing – review & editing

Ahzyris Gibbs: investigation, writing – review & editing

Luz Messina: investigation, methodology, project administration, resources

Mina Halpern: conceptualization, project administration, resources, writing – review & editing

Silvia Amesty: conceptualization, methodology, supervision, writing – review & editing

Click here to access/download
Supporting Information
STROBE Checklist Submission.docx

Click here to access/download
Other
IRB Approval Letter.pdf

Click here to access/download
Other
CONABIOS Letter.PDF
