# Supplementary material for: Thoughts of self-harm in adolescents: Relationship with violence in the Dominican Republic
Source: PLOS Glob Public Health. 2024 Jan 8;4(1):e0002711. doi: 10.1371/journal.pgph.0002711 (PMC10773958; doi:10.1371/journal.pgph.0002711)
Supplement: Attachment — Submitted filename: Response to Reviewers.docx [file pgph.0002711.s006.docx]

Dear Reviewers,

Thank you for your time reviewing our manuscript. We appreciate your thoughtful response and suggestions for edits. We have addressed your concerns and comments below.

First, I would like to address the concern that was raised about our consent process. Thank you for bringing to our attention that we could have explained our rationale better. We decided to waive parental consent for our study because of the sensitivity of the subject matter of our surveys, which asked about each adolescent’s experience with violence. We thought that requiring parental consent would limit participation, especially for those that were most vulnerable. In studies that deal with sensitive subject matter in adolescents, obtaining parental consent has been associated in lower participation and biased samples.^1,2^ Furthermore, the Common Rule (45-CFR-46) permits waivers of parental consent for minimal risk studies under some circumstances.^3^ When this consent process was designed, we balanced our interest in protecting minors with our desire to let the voices of the most vulnerable be heard. We have revised our manuscript to address these concerns.

We have also addressed the concern about the development and validity of the survey in our manuscript. The survey was adopted from surveys the clinic had used in previous studies to estimate the incidence of violence in different clinic populations. Therefore, it was developed and validated within the community in which survey administration took place. We have edited our manuscript to address this concern. Please find the rest of the comments addressed below:

| Comment | Response |
| --- | --- |
| 1. Please include a complete copy of PLOS’ questionnaire on inclusivity in global research in your revised manuscript. Our policy for research in this area aims to improve transparency in the reporting of research performed outside of researchers’ own country or community. The policy applies to researchers who have travelled to a different country to conduct research, research with Indigenous populations or their lands, and research on cultural artefacts. The questionnaire can also be requested at the journal’s discretion for any other submissions, even if these conditions are not met.  Please find more information on the policy and a link to download a blank copy of the questionnaire here: <https://journals.plos.org/globalpublichealth/s/best-practices-in-research-reporting>. Please upload a completed version of your questionnaire as Supporting Information when you resubmit your manuscript. | Thank you, we have uploaded a completed questionnaire with our revised manuscript. |
| 2. Our staff editors have determined that your manuscript is likely within the scope of our Global Mental Health: challenges, opportunities, and the future of the field. This editorial initiative is headed by a team of Guest Editors for PLOS GPH: Rochelle Burgess (University College of London) and Dixon Chibanda (University of Zimbabwe and London School of Tropical Medicine and Hygiene). The Collection invites researchers to submit original research which engages with, or disrupts, the urgent needs across the global mental health landscape. We especially encourage submissions of studies that critically interrogate the status quo of the field and that involve inter-/trans-disciplinary approaches and those which share perspectives from underrepresented global regions and communities.  Additional information can be found on our announcement page: <https://collections.plos.org/call-for-papers/global-mental-health-opportunities-challenges/>  If you would like your manuscript to be considered for this collection, please let us know in your cover letter and we will ensure that your paper is treated as if you were responding to this call.  Please note that being considered for the Collection does not require additional peer review beyond the journal’s standard process and will not delay the publication of your manuscript if it is accepted by PLOS GPH. If you would prefer to remove your manuscript from collection consideration, please specify this in the cover letter | We appreciate you mentioning this. We would like to confirm that we would like to manuscript to be considered for the call for papers “Global Mental Health: Opportunities and Challenges”. We have made this change to in our cover letter. |
| 3. Please ensure that Funding Information and Financial Disclosure Statement are matched. | We have amended our Financial Disclosure statement to read “This project was funded by the Columbia University Vagelos College of Physicians and Surgeons Scholarly Projects program and the Friedman award.  We have no known conflicts of interest to disclose.” Which matches our Funding information. |
| 4. In the Funding Information you indicated that no funding was received. Please revise the Funding Information field to reflect funding received. | This is reflected in the above response. |
| 5. We have noticed that you have uploaded Supporting Information files, but you have not included a list of legends. Please add a full list of legends for your Supporting Information files after the references list. | A full list of legends has been included in the manuscript for the Supporting Information files. |
| 6. In the online submission form, you indicated that "Dataset for this article will become available upon reasonable request". All PLOS journals now require all data underlying the findings described in their manuscript to be freely available to other researchers, either 1. In a public repository, 2. Within the manuscript itself, or 3. Uploaded as supplementary information. This policy applies to all data except where public deposition would breach compliance with the protocol approved by your research ethics board. If your data cannot be made publicly available for ethical or legal reasons (e.g., public availability would compromise patient privacy), please explain your reasons by return email and your exemption request will be escalated to the editor for approval. Your exemption request will be handled independently and will not hold up the peer review process, but will need to be resolved should your manuscript be accepted for publication. One of the Editorial team will then be in touch if there are any issues. | I apologize for the confusion. We will upload the full dataset as a Supporting Information file. |
| Reviewer #1: My question to author is how would you justify the age of adolescents? According to WHO, 10-19 age groups are considered as adolescent. Please clarify your sampling technique. What measures were taken for those having thoughts of harming themselves and others? | Thank you for your feedback. Apologies for the confusion. I agree that the WHO defines adolescence as age 10-19. Our intended target population for this study was those in middle to late adolescence. We have amended our inclusion criteria to state middle to late adolescence to better reflect our population. This population was selected as while early adolescence marks the beginning of puberty, most social and emotional changes occur during middle and late adolescence, as found in the literature.^4,5^  As stated on line 186-187 of the updated manuscript, psychological support services were available at the clinic for participants that were interested. |
| Reviewer #2: Line number 137 to 138 please provide the IRC unique ID number and date of the IRC date taken on the bivariate and the sub group analysis please notify that which statistical test was used (t test,chi-square,fisher exact or other ) | We are sorry, but none of our team is familiar with IRC unique ID number. We would be grateful if you could tell us more about what this means so we could include it in our paper.  We have noted on line 196-201 of the updated manuscript that Chi-squared, Fisher’s exact test, and two sample t-tests were used when appropriate. |

Thank you again for your thoughtful review of our paper. We look forward to hearing back from you.

Sincerely,

Kelsey Badger

References cited in responses:

1. Bauman LJ, Mellins CA, Klitzman R. Whether to Waive Parental Permission in HIV Prevention Research Among Adolescents: Ethical and Legal Considerations. J Law Med Ethics. 2020 Mar;48(1):188-201. doi: 10.1177/1073110520917010. PMID: 32342775; PMCID: PMC8367279.
2. Liu C, Cox RB Jr, Washburn IJ, Croff JM, Crethar HC. The Effects of Requiring Parental Consent for Research on Adolescents' Risk Behaviors: A Meta-analysis. J Adolesc Health. 2017 Jul;61(1):45-52. doi: 10.1016/j.jadohealth.2017.01.015. Epub 2017 Mar 28. PMID: 28363714.
3. Federal Policy for the Protection of Human Subjects (‘Common Rule’) [Internet]. 13 Dec 2022 [cited 13 Aug 2023]. Available from: https://www.hhs.gov/ohrp/regulations-and-policy/regulations/common-rule/index.html.
4. Christie D, Viner R. Adolescent development. BMJ. 2005 Feb 5;330(7486):301-4. doi: 10.1136/bmj.330.7486.301. PMID: 15695279; PMCID: PMC548185.
5. Adolescent and Young Adult Health. World Health Organization [Internet]. 28 April 2023 [cited 13 Aug 2023]. Available from: https://www.who.int/news-room/fact-sheets/detail/adolescents-health-risks-and-solutions.
