# Supplementary material for: Thoughts of self-harm in adolescents: Relationship with violence in the Dominican Republic
Source: PLOS Glob Public Health. 2024 Jan 8;4(1):e0002711. doi: 10.1371/journal.pgph.0002711 (PMC10773958; doi:10.1371/journal.pgph.0002711)
Supplement: Attachment — Submitted filename: Thoughts of self-harm in adolescents_Review.docx [file pgph.0002711.s007.docx]

Thank you for the opportunity to review this interesting manuscript. I believe it is an interesting manuscript with a clear value-add to the field, however, significant revisions are necessary. The most pressing concern is that the statistical analyses are not clear or are inappropriate for the small sample size. Secondly, more information is required to situate these findings in the Dominican Republic in terms of how they relate to culture and other research from the region.

Introduction: Overall, I would suggest revising the structure of this section so the reader is guided to your own research, i.e. P1: the issue (violence against adolescents), P2: violence against adolescents and mental health implications, P3: violence against adolescents and mental health implications in Middle Income Countries, Latin American Countries, or the Dominican Republic if evidence is available. P4: Introducing this study.

- Line 80-81, the first sentence requires minor revisions (adolescence not adolescents) and impacts, not impact. Please note that this issue with adolescence vs adolescents occurs a few times in the manuscript.
- Line 87-91: First, the general literature about violence in childhood and adolescents, and its impact on mental health is discussed in this paragraph. Then there are more specific statistics about the Dominican Republic. I would suggest separating statistics about the Dominican Republic into it’s own paragraph later in the introduction in order to better situate the reader to the context.
- Line 98-99: This sentence should be restructured to help guide the reader. Currently “self-harm thoughts have been linked with bullying and violence against adolescents” implies causality – that self-harm leads to bullying. Please clarify if this is what you mean or revise the sentence.

Methods:

- Line 125-127: “…were recruited by educators from the clinic’s adolescent program.” Can you please clarify how you addressed bias in recruitment? How were participants selected and was there no blinding among recruiters or participants? How might this selection process generate bias in study findings?
- Line 127-128: “The target population for the study was those in middle and late adolescents (note: this should be adolescence) since most social and emotional changes occur during this stage of adolescence” This belongs in the introduction. If you are making an argument that addressing this issue among adolescents is importance, than state that clearly in the introduction rather than the methods section where you are simply stating what was done.
- Line 136: Please write more about survey adaptations. Where previous surveys for organizational assessment purposes or research purposes and what was adapted for this one?
- Analysis: How was sample size determined?
- Line 174: Did you disaggregate the data by different demographic characteristics or just looked at the descriptive characteristics for each category?
- Line 180: “suffering by each type of violence”. I would suggest “experiencing” each type of violence.
- Line 182: Please clarify where Chi-squared tests, Fisher’s exact test, and two-sample t-tests were used. Please note that the Chi-square test is sensitive to sample size, scholars recommend that it is not used if the sample size is less than 50. Why were different tests used here?
- Line 190: “had not yet to complete” should be “had not yet completed”

Results: please clarify where you used the different statistical analysis and why. I am not quite sure why you didn’t just analyze using a Fishers exact test due to the small sample size.

Discussion: Please discuss stigma specifically within the Dominican Republic, how does culture relate to gender norms, relationship violence, and mental health treatment seeking?

Thank you again for the opportunity to review this manuscript, I wish you the best with these revisions and potential future acceptance!
