# Supplementary material for: Thoughts of self-harm in adolescents: Relationship with violence in the Dominican Republic
Source: PLOS Glob Public Health. 2024 Jan 8;4(1):e0002711. doi: 10.1371/journal.pgph.0002711 (PMC10773958; doi:10.1371/journal.pgph.0002711)
Supplement: Attachment — Submitted filename: Response to Reviewers.docx [file pgph.0002711.s008.docx]

Dear Reviewer,

Thank you for your time reviewing our manuscript. We appreciate your thoughtful response and suggestions for edits. We have addressed your concerns and comments below.

We have amended our manuscript to more accurately reflect the statistical analysis that we performed, which we changed our results section so that it reflects analysis done using Fisher’s exact to test for statistical significance. Although literature on the Dominican Republic specifically is difficult to find, we included more background on culture in the Caribbean to give our research more context.

| Comment | Response |
| --- | --- |
| Introduction: Overall, I would suggest revising the structure of this section, so the reader is guided to your own research, i.e. P1: the issue (violence against adolescents), P2: violence against adolescents and mental health implications, P3: violence against adolescents and mental health implications in Middle Income Countries, Latin American Countries, or the Dominican Republic if evidence is available. P4: Introducing this study. | Thank you for all of your comments. We have re-restructured the entire introduction, following a structure of: p1: Violence against adolescents (from line 71 to line 76) *P2:* Effects of violences (line 76 to 86) P3: Estimates per country and previous results from the literature (line 87 to 107) P4: introducing this study (line 108 to 112) |
| Line 80-81, the first sentence requires minor revisions (adolescence not adolescents) and impacts, not impact. Please note that this issue with adolescence vs adolescents occurs a few times in the manuscript. | Thank you for bringing this to our attention. We have performed a deep revision of the language in this article to ensure its clarity about the concepts discussed. |
| Line 87-91: First, the general literature about violence in childhood and adolescents, and its impact on mental health is discussed in this paragraph. Then there are more specific statistics about the Dominican Republic. I would suggest separating statistics about the Dominican Republic into it’s own paragraph later in the introduction in order to better situate the reader to the context. | All of the information found from DR is separated into its own section of the literature review paragraph (line 100 to 107):  *One study estimated the rate of bullying in the DR at 44%,^21^ where bullying was defined as unwanted, aggressive behavior, which involves a real or perceived social power imbalance and is repeated over time.^22^ In the Dominican Republic (DR), it has been estimated that around 80% of adolescents have experienced at least one ACE, with the most prevalent being physical abuse and witnessing domestic violence in their household.^23^ This study also showed that in Dominican adolescents, having experienced more ACEs was associated with depression, anxiety, and dating violence perpetration and victimization.* |
| Line 98-99: This sentence should be restructured to help guide the reader. Currently “self-harm thoughts have been linked with bullying and violence against adolescents” implies causality – that self-harm leads to bullying. Please clarify if this is what you mean or revise the sentence. | We have changed this line to establish relation rather than causation (now in line 84):  *Specifically, self-harm thoughts have been potentially associated with bullying and violence against adolescents,^8^ and they are associated with increased risk of suicidal ideation and suicide completion.* |
| Line 125-127: “…were recruited by educators from the clinic’s adolescent program.”  Can you please clarify how you addressed bias in recruitment?  How were participants selected and was there no blinding among recruiters or participants?  How might this selection process generate bias in study findings? | Further details have been written about the recruitment process (line 119):  *A total of 49 participants aged 13-20 and residents of La Romana, DR were recruited by educators using a two-pronged approach: one part of the recruitment took place during the educator’s sessions with adolescents. In that sense, sampling was convenient sampling in nature. To include the adolescents who did not have access to CFLR educators, we also encouraged adolescents to refer their peers to complete the survey.*  As added in line 124: *Participants were not blinded nor randomize due to the small sample for the study.*  We have added the following section to our discussion section (line 342):  *Furthermore, our recruitment process also limits our results, in that we performed recruitment mainly through CFLR educators. To reduce this bias, we encouraged adolescents to refer their peers who did not have contact with CFLR educators, and invite them to participate in the survey. Even so, a limitation in our study is that we cannot ensure generalizability, since the experiences of those who were not willing or able to participate may be different from the adolescents who did participate in the study.* |
| Line 127-128: “The target population for the study was those in middle and late adolescents (note: this should be adolescence) since most social and emotional changes occur during this stage of adolescence” This belongs in the introduction. If you are making an argument that addressing this issue among adolescents is importance, than state that clearly in the introduction rather than the methods section where you are simply stating what was done. | Thank you for your suggestion, we have changed this sentence and included it in the introduction. |
| Line 136: Please write more about survey adaptations. Where previous surveys for organizational assessment purposes or research purposes and what was adapted for this one? | Further details were added about the survey creation in line 133:  *The survey was adapted from surveys the research department at the clinic designed and used in previous studies to estimate incidence of violence in other populations. Furthermore, the survey was pre-tested on small groups of adolescents to ensure clarity and cultural appropriateness, and small changes were made to survey wording and flow to ensure adolescents’ understanding of each question posed. This ensured that we kept the most relevant questions to the Dominican adolescent population.* |
| Analysis: How was sample size determined? | While we would have been thrilled to have a larger sample size, our resources were limited in conducting this study. Our sample size is the largest we could accommodate given our limited resources. |
| Line 174: Did you disaggregate the data by different demographic characteristics or just looked at the descriptive characteristics for each category? | We looked at the descriptive characteristics for each category. This has now been clarified in Methods (line 173):  *We began analysis by doing descriptive statistic of each demographic category and violent event reported.* |
| Line 180: “suffering by each type of violence”. I would suggest “experiencing” each type of violence. | Thank you for noticing this. We have made this correction in our manuscript. |
| Line 182: Please clarify where Chi-squared tests, Fisher’s exact test, and two-sample t-tests were used. Please note that the Chi-square test is sensitive to sample size, scholars recommend that it is not used if the sample size is less than 50. Why were different tests used here? | I appreciate you bring this to our attention. Upon conferring among authors, we agree that Fisher’s exact test is a better test given our diminished population and ran our analysis with Fisher’s exact test alone when comparing categorical variables and two-sample t-test was used when comparing two means. This has been clarified in our manuscript. |
| Line 190: “had not yet to complete” should be “had not yet completed.” | This has been corrected. |
| Results: please clarify where you used the different statistical analysis and why. I am not quite sure why you didn’t just analyze using a Fishers exact test due to the small sample size. | Thank you for this observation. This has been clarified in the Methods section and our Results now reflect the use of Fisher’s exact test only (line 182):  *For bivariable analysis, we used Fisher’s exact test (when appropriate), and two-sample t-test (when comparing two means) for measuring association. A value of p < 0.05 was set for statistical significance. The associations were test between having self-harm thoughts, participant demographics and experiencing violence.* |
| Discussion: Please discuss stigma specifically within the Dominican Republic, how does culture relate to gender norms, relationship violence, and mental health treatment seeking? | We were not able to find literature or news articles on stigma about mental health services specifically for the DR. However, since this comment goes beyond the scope of our research objective, we cannot infer any stigma about seeking healthcare from our results. |

Thank you again for your thoughtful review of our paper. We look forward to hearing back from you.

Sincerely,

Kelsey Badger
